# Deep Reinforcement Learning of Mobile Robot Navigation in Dynamic Environment: A Review

**DOI:** 10.3390/s25113394

**Published:** 2025-05-28

**Authors:** Yingjie Zhu, Wan Zuha Wan Hasan, Hafiz Rashidi Harun Ramli, Nor Mohd Haziq Norsahperi, Muhamad Saufi Mohd Kassim, Yiduo Yao

**Affiliations:** Department of Electrical and Electronic Engineering, Faculty of Engineering, Universiti Putra Malaysia, Serdang 43400, Selangor, Malaysiahrhr@upm.edu.my (H.R.H.R.); nmhaziq@upm.edu.my (N.M.H.N.); saufi@upm.edu.my (M.S.M.K.);

**Keywords:** deep reinforcement learning, dynamic environments, mobile robot, navigation

## Abstract

Deep reinforcement learning (DRL), a vital branch of artificial intelligence, has shown great promise in mobile robot navigation within dynamic environments. However, existing studies mainly focus on simplified dynamic scenarios or the modeling of static environments, which results in trained models lacking sufficient generalization and adaptability when faced with real-world dynamic environments, particularly in handling complex task variations, dynamic obstacle interference, and multimodal data fusion. Addressing these gaps is essential for enhancing its real-time performance and versatility. Through a comparative analysis of classical DRL algorithms, this study highlights their advantages and limitations in handling real-time navigation tasks under dynamic environmental conditions. In particular, the paper systematically examines value-based, policy-based, and hybrid-based DRL methods, discussing their applicability to different navigation challenges. Additionally, by reviewing recent studies from 2021 to 2024, it identifies key trends in DRL-based navigation, revealing a strong focus on indoor environments while outdoor navigation and multi-robot collaboration remain underexplored. The analysis also highlights challenges in real-world deployment, particularly in sim-to-real transfer and sensor fusion. Based on these findings, this paper outlines future directions to enhance real-time adaptability, multimodal perception, and collaborative learning frameworks, providing theoretical and technical insights for advancing DRL in dynamic environments.

## 1. Introduction

Since the mid-20th century, robotics has undergone rapid development, evolving from simple industrial automation devices to complex intelligent autonomous robots. Initially, robots were primarily employed in industrial production to perform highly repetitive mechanized tasks, aiming to enhance production efficiency and reduce manual labor [1]. However, with significant advancements in computational power, particularly the development of integrated circuits, and the gradual refinement of sensor technologies, robots have moved beyond preprogrammed operations and basic mechanical actions to acquire capabilities such as environmental perception, autonomous decision-making, and adaptability [2]. Today, the application of robotics has extended from traditional industrial production to various fields, including healthcare, logistics, services, and agriculture, making it an indispensable technological support across multiple industries [3].

In autonomous robot navigation, path planning and simultaneous localization and mapping (SLAM) are two core challenges [4]. Path planning involves providing robots with the optimal route from a starting point to a target destination [5], considering factors such as obstacle avoidance, safety, path length, and time efficiency. Traditional path planning algorithms, such as the A* algorithm, Dijkstra’s algorithm, and rapidly exploring random tree (RRT), perform well in static and well-known environments, systematically searching for globally optimal solutions [6]. Despite this, in dynamic, complex, or unknown environments, the limitations of these methods become increasingly apparent. Obstacles and target positions in dynamic environments frequently change, requiring traditional algorithms to replan paths repeatedly, which not only increases computational overhead but also compromises real-time performance [7,8,9,10,11]. Furthermore, these methods lack sufficient adaptability to address environmental uncertainties and dynamic changes, making them inadequate for meeting the demands of complex scenarios [12].

DRL, as an emerging technology combining deep learning and reinforcement learning, offers a novel approach to robot navigation. DRL leverages deep neural networks to extract meaningful features from raw sensor data and generates optimized decision strategies by autonomously learning the dynamic characteristics of complex environments [13]. Compared to traditional methods, DRL not only adapts quickly to environmental changes but also enables real-time planning in large-scale dynamic environments, significantly improving the efficiency and accuracy of path planning. In recent years, DRL has been widely applied in areas such as unmanned vehicles, industrial automation, and robot navigation in dynamic scenarios, becoming a hotspot in current research [14].

**Figure 1:**
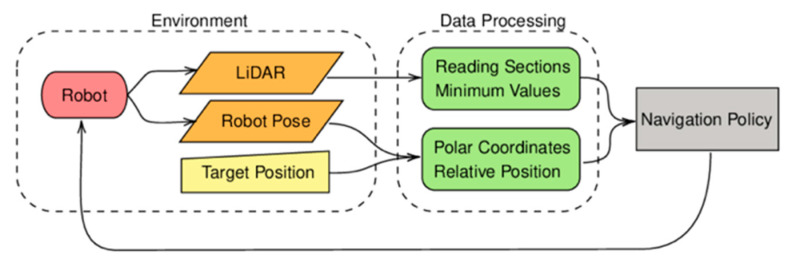
Proposed navigation system overview [17].

Despite the immense potential of DRL applications in dynamic environments, current research still faces significant challenges. Many models are trained in relatively simple environments, lacking comprehensive simulation of dynamic and complex scenarios, which limits the generalization capability of DRL models. This limitation becomes particularly pronounced when addressing complex scenarios involving dynamic obstacles, random target changes, and environmental disturbances [15]. When robots navigate in new environments, strategies trained only in static environments struggle to effectively handle complex dynamic changes, potentially leading to decreased success rates in path planning or even erroneous decisions. To address this issue, researchers have recently proposed training DRL models in dynamic environments, incorporating more complex and diverse dynamic scenarios to enable robots to learn how to adapt to environmental changes and adjust strategies rapidly [16]. This training approach significantly enhances the robustness and adaptability of DRL, laying a solid foundation for efficient robot navigation in practical applications. This special relationship is shown in Figure 1.

**Figure 2:**
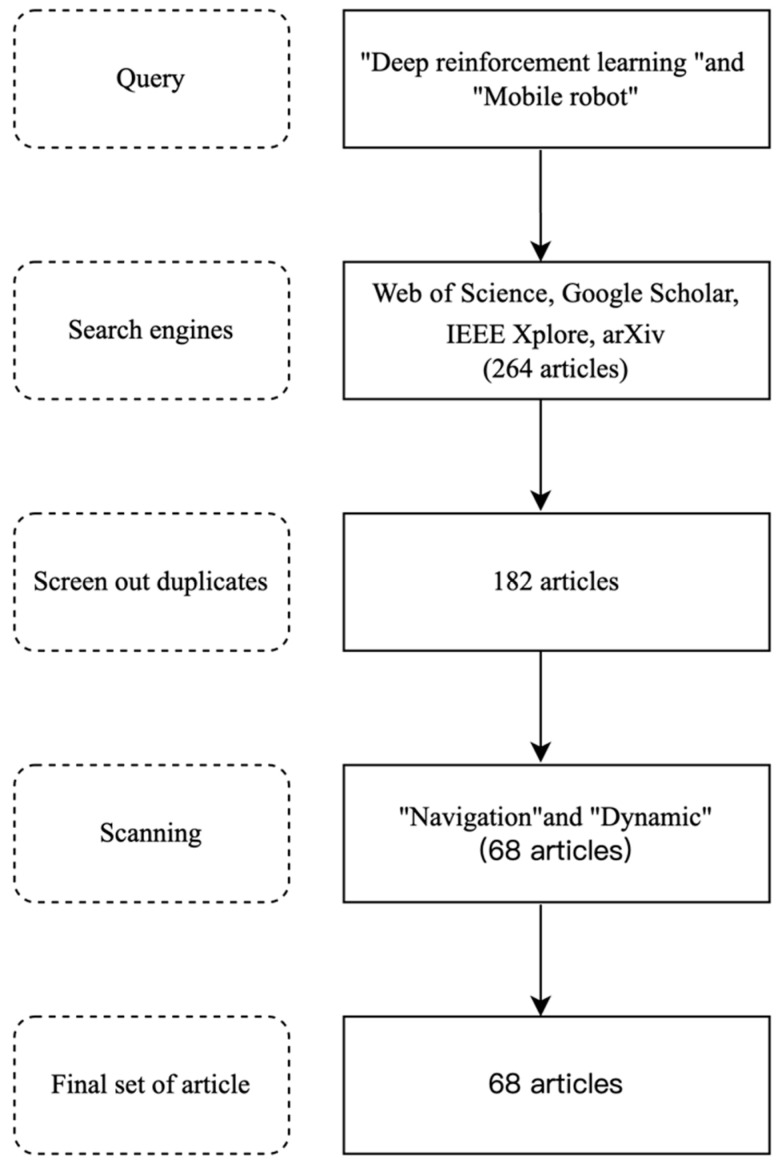
Selection of study, search query, and inclusion criteria.

To comprehensively understand the development status of DRL technology in dynamic environments, this study included a systematic review of recent research results based on the Web of Science, Google Scholar, IEEE Xplore, and arXiv database. The review covers relevant literature published between 2021 and 2024, with keywords including “dynamic environment”, “deep reinforcement learning”, and “mobile robot navigation”. Through extensive literature screening and analysis, this study summarizes the latest advancements in DRL technology for path planning in dynamic environments, as shown in Figure 2.

These studies span various aspects, from algorithm design to practical applications, revealing the core trends in current technological development and their potential applications in dynamically complex scenarios [18]. Additionally, this study systematically outlines the main challenges faced by DRL technology in addressing complex dynamic scenarios, such as real-time performance [19], generalization capability, and robustness to environmental changes. Based on this analysis, the study not only discusses the current state of DRL applications in dynamic environments but also delves into future research directions, aiming to provide valuable theoretical support and technical guidance for researchers in this field [20].

In recent years, DRL has attracted significant attention in the field of mobile robot navigation, leading to numerous systematic review studies. To provide a comprehensive understanding of the current research landscape, recent review articles on the applications, challenges, and development trends of DRL in mobile robot navigation have been examined. The key findings are summarized in Table 1.

This paper systematically reviews the applications of DRL in mobile robot navigation within dynamic environments, with a particular focus on key technological developments in environmental adaptability, multimodal perception fusion, and task scene diversity. Unlike previous studies that primarily focus on algorithmic improvements, this paper emphasizes the integration of DRL with multimodal perception and real-time planning, aiming to bridge the gap between theoretical advancements and practical implementations. Through a comparative analysis of value-based, policy-based, and hybrid DRL methods, this study highlights their advantages and limitations in handling real-time navigation tasks under dynamic environmental conditions. Additionally, the paper identifies critical challenges, including the generalization capability of DRL models in unseen environments, policy robustness in the face of dynamic obstacle interference, and the efficiency of sim-to-real transfer for practical deployment. Based on these findings, future research directions are proposed, emphasizing improvements in real-time adaptability, scalable multimodal data fusion techniques, and the development of collaborative DRL frameworks for multi-robot navigation. These insights provide a theoretical foundation and technical reference for enhancing DRL’s effectiveness in complex and dynamic real-world scenarios. For example, in dynamic environments, robots often encounter complex scenario changes, including moving obstacles, uncertainties in target positions, and the need for efficient fusion of multimodal perception data [27]. To address these issues, this study summarizes recent optimization solutions and proposes future improvement directions. In particular, this study analyzes the innovations in cutting-edge research and discusses their impact on practical applications, especially in enhancing adaptability in dynamic environments, improving multimodal perception fusion capabilities, and achieving the transfer from simulation to real-world applications. Furthermore, the study highlights the potential of DRL technology in improving the robustness and safety of algorithms, pointing out that safe and efficient navigation in complex dynamic environments will become a crucial objective of future research [28]. By thoroughly examining these issues, this paper aims to provide theoretical foundations and technical references for the field of robot navigation and to open new avenues for future research.

## 2. Theories and Comparisons of Deep Reinforcement Learning Methods

DRL, as a significant branch of artificial intelligence, has emerged as a key technology for solving tasks such as navigation and control in dynamic environments due to its ability to learn in high-dimensional state spaces and its strong adaptability to complex decision-making problems [13]. By combining the decision-making optimization mechanism of reinforcement learning with the nonlinear feature representation capability of deep neural networks, DRL has successfully overcome the scalability bottlenecks of traditional reinforcement learning methods in complex environments [18]. In recent years, researchers have developed various DRL approaches tailored to the requirements of different task scenarios, including value-based methods, policy-based methods, and hybrid methods. These approaches have made significant progress in addressing issues such as overestimation bias, computational complexity, and policy robustness [16,29], laying a theoretical and practical foundation for further expanding the application scope of DRL technologies.

This paper systematically summarizes the development and applications of DRL methods from two dimensions: theoretical framework and technical comparisons. First, it introduces the core theories of DRL, including its modeling approach based on Markov decision processes (MDPs) [30] and policy optimization mechanisms, and outlines the main ideas and characteristics of value-based, policy-based, and hybrid methods. Subsequently, through a comparative analysis of classical DRL algorithms, this paper summarizes the differences in their performance in complex environments and their applicable scenarios, further exploring their advantages and limitations in practical applications.

### 2.1. Theoretical Framework of Deep Reinforcement Learning

Reinforcement learning (RL) is a machine learning paradigm that aims to enable an agent to learn an optimal policy for maximizing long-term cumulative rewards through interactions with its environment [18]. In the traditional RL framework, problems are modeled using MDPs, which consist of states (S), actions (A), rewards (R), and state transition probabilities (P) [30]. The agent selects actions based on a policy (π) and optimizes decision-making behavior according to the reward function. Nonetheless, traditional RL methods often struggle to scale when confronted with high-dimensional state spaces and complex dynamic environments [31].

DRL combines deep learning with reinforcement learning by leveraging deep neural networks (DNNs) to approximate the state-action space, effectively addressing the computational bottlenecks of high-dimensional state spaces [32]. DRL methods have demonstrated robust performance across various domains, such as robotic control, game agents, and autonomous driving, becoming an essential tool for solving complex decision-making problems.

The development of DRL can be broadly categorized into three directions: value-based methods, policy-based methods, and hybrid methods. Value-based methods indirectly derive the optimal policy by learning the state-action value function [33]; policy-based methods directly optimize the policy function and are particularly suited for continuous action spaces [29]; and hybrid methods combine the strengths of both approaches to more efficiently handle complex tasks [34]. These methods not only reflect the technical advancements of DRL but also provide flexible solutions for addressing various types of tasks. Value-based methods, such as the deep Q-network (DQN) [13], have demonstrated outstanding performance in gaming tasks, particularly in Atari games. Hybrid methods, such as the deep deterministic policy gradient (DDPG) [16] and twin delayed deep deterministic policy gradient (TD3) [35], have shown exceptional effectiveness in robotic continuous control tasks, as they are capable of learning precise control strategies for high-dimensional continuous action spaces in a stable manner.

In recent years, research in DRL has gradually shifted from theoretical modeling to practical applications. Classical algorithms such as DQN, proximal policy optimization (PPO), and soft actor-critic (SAC) have not only undergone continuous optimization but also addressed critical challenges such as overestimation bias, computational complexity, and exploration efficiency [29,36,37]. The innovations in these methods have established a foundation for the development of DRL in multiple fields and have led the way for future research.

### 2.2. Comparison of DRL with Classical and Other Learning-Based Navigation Methods

Before conducting an in-depth analysis of different DRL methods, it is essential to first compare DRL with other commonly used navigation approaches to provide a more comprehensive perspective. Classical control methods, including Dijkstra’s algorithm, A*, RRT, and the dynamic window approach (DWA), have long been employed for robot navigation due to their deterministic properties and well-defined mathematical foundations. These methods excel in structured and static environments, offering precise path planning with minimal computational requirements. However, their performance deteriorates in highly dynamic scenarios, as they require frequent re-planning and struggle with real-time adaptability [38]. In contrast, DRL enables robots to learn adaptive policies through continuous interaction with the environment, allowing real-time decision-making and improved generalization in non-stationary settings [39]. Nevertheless, DRL’s training complexity and reliance on extensive computational resources, as well as the necessity of well-designed reward functions, pose significant challenges [40]. To provide a more comprehensive comparison of different navigation approaches, Table 2 summarizes the key characteristics, advantages, disadvantages, and applicable scenarios of classical control methods, DRL, hybrid learning approaches, and multi-agent reinforcement learning (MARL).

Beyond classical control, hybrid learning-based approaches such as Neuro-SLAM and imitation learning have been explored to enhance navigation efficiency. Neuro-SLAM integrates deep learning with SLAM, leveraging neural networks to refine perception and improve localization accuracy [48]. While it enhances mapping capabilities, its reliance on pre-trained models may limit adaptability to previously unseen environments [49]. Imitation learning, on the other hand, enables robots to mimic expert demonstrations, facilitating faster learning and reducing the need for explicit reward engineering in reinforcement learning [50]. However, its dependency on high-quality expert data makes it difficult to generalize beyond pre-collected scenarios, contrasting with DRL’s ability to autonomously optimize policies through trial-and-error learning [51].

MARL extends DRL’s capabilities to collaborative multi-robot navigation, where multiple agents interact within the same environment to achieve coordinated objectives. Unlike single-agent DRL, MARL allows robots to exchange information, adapt to dynamic teammates or adversaries, and develop cooperative strategies [52]. While MARL significantly enhances multi-robot efficiency in applications such as swarm navigation and task allocation, it also introduces additional challenges, including communication constraints, increased computational complexity, and the difficulty of achieving policy convergence in large-scale systems.

Comparing these approaches highlights the trade-offs between different navigation strategies. Classical control methods provide strong theoretical guarantees but suffer from poor adaptability in dynamic settings, whereas DRL enhances decision-making flexibility at the cost of increased training complexity. Hybrid learning methods bridge perception and control but often require domain-specific optimization, while MARL expands reinforcement learning to collaborative scenarios but faces scalability issues [53]. These comparisons illustrate the need for integrating multiple approaches to fully exploit their advantages and enhance robot navigation in complex and dynamic environments.

While DRL provides flexible and adaptive strategies for robot navigation, it is important to recognize that not all navigation problems require or benefit from DRL-based solutions. In practice, the suitability of DRL depends heavily on task complexity, environmental dynamics, computational constraints, and safety requirements. To provide additional context for the following algorithm-specific discussions, Table 3 summarizes typical navigation scenarios and assesses whether DRL is appropriate, based on representative task characteristics.

**Table 3:** DRL suitability for common navigation problems.

Ref.	Problem Type	Typical Scenario	DRL Suitability	Rationale
[54]	Static path planning	Indoor warehouse with fixed layout	Not preferred	Traditional planners (A*, Dijkstra) are fast and optimal
[55]	Dynamic obstacle avoidance	Urban sidewalk with moving pedestrians	Suitable	DRL adapts to real-time interactions and uncertainties
[56]	Multi-agent coordination	Multi-robot logistics in shared space	Suitable	DRL enables decentralized adaptive policies
[57]	Deterministic rules and known map	Factory floor with preprogrammed signals	Not preferred	Rule-based FSMs are reliable, interpretable, and efficient
[58]	Sensor-constrained, low-latency tasks	Drone hovering in tight indoor space	Not preferred	DRL inference may exceed timing constraints; PID or MPC preferable
[59]	Unstructured outdoor exploration	Off-road navigation with unknown terrain	Suitable	DRL handles partial observability and long-term reasoning

As summarized above, DRL demonstrates particular strengths in managing complex, uncertain, and dynamic scenarios. The next section presents a comparative analysis of representative DRL algorithms, focusing on their theoretical characteristics and performance trade-offs across such scenarios.

### 2.3. Comparison of Classical DRL Methods

DRL, as an intelligent technology combining the strengths of deep learning and reinforcement learning, has achieved remarkable success in addressing complex decision-making problems. However, different DRL algorithms emphasize distinct aspects in their design, each possessing unique advantages and corresponding limitations. A comparative analysis of classical DRL methods provides a comprehensive understanding of their applicable scenarios and constraints, offering guidance for algorithm selection in practical applications. Table 4 summarizes the main characteristics of several classical DRL algorithms, including value-based, policy-based, and hybrid methods.

Value-based methods learn optimal policies by estimating state-action value functions, characterized by high learning efficiency and stability. Despite this, they may encounter issues such as overestimation bias and scalability in complex environments. DQN, proposed by Mnih et al., is a pioneering method in this category. It combines deep neural networks with experience replay to address the scalability challenges of traditional Q-learning in high-dimensional state spaces. DQN also introduces target networks to enhance learning stability. Nevertheless, it is prone to overestimation bias in Q-value estimation and exhibits high sensitivity to hyperparameters [33].

To mitigate the overestimation bias of DQN, Hasselt et al. proposed the double deep Q-network (double DQN), which separates action selection from value estimation, significantly reducing bias and improving policy stability [60]. Despite its improved accuracy, double DQN increases computational complexity due to maintaining two Q-networks. Subsequently, Wang et al. introduced the dueling deep Q-network (dueling DQN), which optimizes Q-value estimation by decomposing it into state value and action advantage to enable more effective evaluation of critical states [60]. However, this improvement introduces additional network architecture complexity and sensitivity to hyperparameter settings.

Policy-based methods directly optimize the policy function, making them particularly suitable for tasks involving high-dimensional and continuous action spaces. Trust region policy optimization (TRPO), proposed by Schulman et al., is a significant advancement in this domain. TRPO ensures policy improvement stability and monotonicity by introducing a Kullback–Leibler (KL) divergence constraint, performing well in high-dimensional action spaces [37,61]. Nonetheless, its high computational complexity limits its applicability in real-time tasks. To simplify the policy optimization process, Schulman et al. proposed PPO, which employs a clipping mechanism to reduce computational costs while achieving a good balance between policy stability and learning efficiency [29]. This makes PPO highly suitable for real-time tasks, although its performance may degrade in highly complex environments and is sensitive to hyperparameters such as the clipping threshold.

Hybrid methods combine the advantages of value-based and policy-based approaches to better address challenges in complex tasks. Asynchronous advantage actor-critic (A3C), proposed by Mnih et al., improves training efficiency through asynchronous updates, eliminating the need for experience replay memory [33]. However, A3C exhibits relatively low sample efficiency and demands significant computational resources. DDPG, proposed by Lillicrap et al., represents another milestone for hybrid methods, excelling in continuous action spaces by leveraging the actor-critic architecture to integrate policy optimization and Q-value estimation [17]. Despite this, DDPG is highly sensitive to hyperparameters and prone to policy overfitting issues.

To further address overestimation bias, Fujimoto et al. proposed TD3 [35]. TD3 employs techniques such as dual Q-networks, delayed policy updates, and target policy smoothing, significantly enhancing training stability and accuracy. Similarly, Haarnoja et al. introduced SAC, which enhances policy exploration through a maximum entropy framework and reduces bias using dual Q-networks. However, both SAC and TD3 increase computational complexity due to the need to train multiple networks and are highly sensitive to hyperparameter adjustments [62].

**Table 4:** Advantages and disadvantages of classic DRL technology.

Category	Ref.	Method	Year	Computation Cost	Sample Efficiency	Robustness to Environmental Changes	Advantages	Disadvantages
Value-Based DRL Methods	[33,58,63]	Deep Q-Network (DQN)	2013	Medium	Low	Low	Addresses the scalability issue of Q-learning by using deep neural networks for function approximation. Stabilizes learning through techniques like experience replay and a target network.	Prone to overestimation bias in Q-value estimation. High computational complexity due to the use of neural networks. Sensitive to hyperparameter settings, such as learning rate and replay buffer size.
	[57,60,64]	Double Deep Q-Network (Double DQN)	2015	Medium	Medium	Medium	Reduces overestimation bias by decoupling action selection and value estimation. Improves stability and accuracy in policy learning compared to DQN.	Increases computational complexity due to maintaining two Q-networks. Sensitive to hyperparameter tuning, especially learning rates and update frequencies.
	[61,65]	Dueling Deep Q-Network (Dueling DQN)	2016	Medium	Medium	Medium	Separates state value and action advantage, enabling more precise Q-value estimation. Enhances learning efficiency by focusing on state evaluation in scenarios with minimal action-value differences.	Increased computational complexity due to the additional network architecture. Sensitive to hyperparameter tuning and network design choices.
Policy-Based DRL Methods	[8,37]	Trust Region Policy Optimization (TRPO)	2015	High	Medium	High	Ensures stable and monotonic policy improvement through trust region constraints. Effective for high-dimensional or continuous action spaces.	Computationally expensive due to solving constrained optimization problems. Complex implementation compared to simpler policy gradient methods.
	[37,66]	Proximal Policy Optimization (PPO)	2017	Medium	High	Medium	Simplifies trust region optimization with a clipping mechanism, improving computational efficiency. Balances policy stability and learning efficiency, making it suitable for real-time tasks.	Sensitive to hyperparameter tuning, particularly the clipping threshold. May still face performance degradation in highly complex environments.
Hybrid-Based DRL Methods	[67,68]	Asynchronous Advantage Actor-Critic (A3C)	2016	Low	Medium	Medium	Improves training efficiency through asynchronous updates from multiple parallel agents. Eliminates the need for experience replay, reducing memory requirements.	Lower sample efficiency compared to methods using experience replay. Requires significant computational resources for parallel processing.
	[16,69]	Deep Deterministic Policy Gradient (DDPG)	2015	Medium	Medium	Low	Handles high-dimensional continuous action spaces effectively. Combines the strengths of policy gradient and value-based methods using an actor-critic framework.	Prone to overfitting and instability due to deterministic policies. Requires extensive hyperparameter tuning and is sensitive to noise settings.
	[36,70]	Soft Actor-Critic (SAC)	2018	High	Medium	High	Encourages exploration with the maximum entropy framework, improving robustness. Reduces overestimation bias using dual Q-networks, enhancing stability.	Computationally intensive due to simultaneous training of multiple networks. Performance is highly sensitive to the entropy weighting coefficient.
	[35,71]	Twin Delayed Deep Deterministic Policy Gradient (TD3)	2018	Medium	Medium	High	Reduces overestimation bias with twin Q-networks for more accurate value estimation. Improves stability with delayed policy updates and target policy smoothing.	Computationally expensive due to training multiple networks. Sensitive to hyperparameter tuning, such as update delays and noise settings.

Note: For visual representations of the algorithms summarized in this table, please refer to Appendix A.

As shown in Table 4, different DRL methods exhibit unique characteristics in terms of computational complexity, learning stability, and policy optimization efficiency. To further enhance the understanding of representative reinforcement learning algorithms, this study presents a systematic performance analysis of value-based, policy-based, and hybrid methods in robot path navigation tasks under dynamic environments. By incorporating empirical results from recent studies, key distinctions in path efficiency, convergence rate, and robustness are examined in greater detail.

Value-based methods, which rely on value function estimation for policy evaluation, have demonstrated varying degrees of effectiveness in navigation tasks. For instance, Arce et al. reported that DQN achieved an average success rate of approximately 86% in indoor mobile robot experiments, with an average path length of 21.3 m [72]. However, policy stability was limited and the model required around 20,000 training steps to converge. Despite their structural simplicity and ease of implementation, value-based methods are constrained by their reliance on discrete action space modeling, making them less suitable for continuous control tasks. To address overestimation bias, double DQN introduces decoupled action selection and evaluation mechanisms, thereby improving convergence stability. Meanwhile, dueling DQN incorporates advantage functions to better capture the importance of actions in complex states. Nevertheless, both methods remain confined to discrete control domains and are best suited for virtual simulations or environments with clearly defined states and transitions [60].

In contrast, policy-based methods, such as PPO, exhibit stronger generalization and adaptability to continuous control scenarios. In a recent study by Reda, PPO achieved a 90% average success rate in dynamic path planning tasks, with the average path length reduced to 18.5 m and convergence achieved within 15,000 steps [73]. These methods offer more flexible learning in high-dimensional action spaces. TRPO, although it enhances policy stability via KL divergence constraints, incurs high computational overhead and lacks real-time applicability in physical robotic platforms. Asynchronous advantage A3C accelerates convergence through parallel multi-threaded training, but its dependence on synchronous environment updates may introduce instability in real-world deployments, making it more suitable for simulation settings [67]. PPO, in particular, strikes a practical balance between training efficiency and policy robustness and has been widely applied in tasks such as path planning and multi-goal obstacle avoidance.

Hybrid methods, which integrate the strengths of both value-based and policy-based approaches, have become a key research direction in DRL for robotics. DDPG, an actor-critic framework, demonstrated a 95% success rate and the lowest path planning error in Cai et al.’s AGV navigation experiments, converging in under 10,000 training steps [74]. SAC, by introducing a maximum entropy objective, enhances policy diversity and exploration, making it particularly effective in highly uncertain environments such as human-crowd navigation. TD3 further improves control stability and convergence reliability through delayed policy updates and twin Q-network structures, and has shown strong performance in multi-goal navigation and robust trajectory tracking tasks [35,36]. Despite these advancements, hyperparameter sensitivity and high training complexity remain key challenges. Future studies should prioritize improvements in algorithmic robustness, computational efficiency, and transferability to better meet the complex requirements of real-world robotic applications.

## 3. Key Technologies of Deep Reinforcement Learning in Dynamic Environment Navigation

With the continuous development of the theoretical framework and practical applications of DRL, its potential in the field of robotic navigation has been widely recognized. However, achieving efficient path planning and decision-making in dynamic environments remains a significant challenge. The DRL theoretical framework and algorithms introduced in Section 2 provide a solid foundation for addressing complex tasks in high-dimensional state spaces. Nevertheless, the inherent characteristics of dynamic environments—such as random variations in obstacles, diverse task scenarios, and the need for integrating complex perception data—place higher demands on the adaptability and robustness of navigation algorithms. Therefore, exploring how to deeply integrate DRL’s perception capabilities, decision optimization, and real-time control to meet the demands of navigation in dynamic environments constitutes a primary objective of this study.

### 3.1. Adaptability and Robustness in Dynamic Environments

The complexity of dynamic environments emphasizes the high demands on adaptability and robustness for navigation algorithms. Random changes in obstacles, uncertainties in target locations, and the stringent requirements of real-time path planning render traditional methods, such as the DWA and RRT, inadequate in dynamic scenarios [6,43]. As an emerging intelligent technology, DRL provides innovative solutions to these challenges [75].

The main sources of complexity in dynamic environments include random obstacle variations, target location uncertainties, and real-time path planning demands. These features challenge navigation algorithms to maintain high decision accuracy while quickly perceiving and responding to changes [76]. Traditional methods like DWA and RRT often fail to meet the dual requirements of real-time performance and handling complex scenarios in dynamic environments [77]. Consequently, DRL has emerged as a promising alternative to tackle navigation issues in such environments [78].

By combining deep learning with reinforcement learning, DRL demonstrates significant advantages in managing dynamic complexity. For instance, Sangiovanni et al. proposed a hybrid control method based on a dynamic switching mechanism that dynamically adjusts obstacle avoidance strategies when obstacles approach, balancing real-time responses with path optimization [11]. This mechanism effectively integrates the rule-based reliability of traditional methods with the adaptability of DRL, providing efficient solutions for complex navigation tasks in dynamic environments. Similarly, Patel et al. designed a dynamic velocity control system by combining DRL and DWA, where DWA quickly generates feasible local avoidance solutions while DRL optimizes the global strategy, enabling flexible responses to dynamic obstacles in complex environments [79].

Enhancing perception and decision-making capabilities is another critical direction for improving navigation performance in dynamic environments. Zhou et al. proposed a DRL algorithm based on heterogeneous graph attention networks (HGATs) to model complex human–robot interaction behaviors [80]. This algorithm focuses on behavioral patterns in crowded environments, significantly improving the robot’s obstacle avoidance and decision-making efficiency. Furthermore, Feng et al. explored the application of multimodal perception in dynamic environments by designing an obstacle avoidance strategy that integrates LiDAR and visual data, optimizing navigation performance in complex scenarios such as narrow corridors [81]. These studies demonstrate that enhancing perception modules’ intelligence can significantly improve DRL’s adaptability and robustness in dynamic environments. The practical applicability of DRL has been verified in real-world scenarios. For example, Wenzel et al. developed an end-to-end visual navigation system that captures obstacle positions and trajectories in real time using monocular vision, enabling dynamic path planning under constrained hardware resources while effectively addressing obstacle occlusion issues [82]. Additionally, Beomsoo et al. designed a DRL algorithm based on 2D LiDAR data, employing a stochastic sampling strategy to accelerate model training and demonstrating superior dynamic obstacle avoidance in industrial environments [83]. These applications further validate DRL’s practical applicability in dynamic environments.

Nevertheless, current methods face challenges such limited generalization capabilities and real-time responsiveness due to the complexity of dynamic scenarios. The high computational cost of integrating multimodal perception data further exacerbates these issues.

### 3.2. Multimodal Perception and Data Fusion

In dynamic environments, robotic navigation systems must handle diverse and complex information from their surroundings, which often exceeds the capabilities of single-sensor systems to ensure sufficient accuracy and robustness. For instance, while LiDAR provides precise distance measurements, it may encounter blind spots in occluded or complex dynamic scenarios. Similarly, visual sensors capture rich environmental features but may struggle with stability under varying lighting conditions. Multimodal perception and data fusion have thus become critical to enhancing the overall performance of navigation systems, enabling robots to integrate data from LiDAR, cameras, ultrasonic sensors, and more, resulting in improved perception accuracy and navigation robustness.

The development of DRL has further advanced the efficient fusion of multimodal perception data. Kaymak et al. developed a dueling double deep Q-network (D3QN) based on multisensor fusion, combining visual and force feedback data [84]. This approach not only improved humanoid robots’ navigation performance in dynamic environments but also enhanced their stability during locomotion. Similarly, Zhang et al. proposed a spatiotemporal DRL method that integrates LiDAR and camera data, which significantly improves the robustness of path-following in industrial logistics scenarios [85]. These studies emphasize the benefits of multimodal perception data fusion in allowing navigation systems to adapt more effectively to complex environments in dynamic scenarios.

However, real-time fusion of multimodal data poses challenges in balancing computational complexity and responsiveness. To address this, Chai et al. proposed a hierarchical deep learning control framework that employs recurrent neural networks (RNNs) at the motion planning layer to predict optimal trajectories and integrates DRL strategies at the obstacle avoidance layer [86]. By combining historical data with real-time sensor inputs, this layered architecture significantly reduces online optimization costs while improving navigation accuracy and real-time performance. Similarly, Liang et al. designed a context-aware DRL framework for mapless navigation. This framework dynamically models the environment state based on multimodal sensor inputs, enabling efficient decision-making in complex scenarios [87]. These approaches underscore the importance of real-time fusion of multimodal perception data for robot navigation in dynamic environments, while also requiring a balance between computational efficiency and environmental adaptability.

Notably, multimodal perception not only enhances navigation performance but also expands robots’ adaptability to unknown environments. By combining LiDAR and visual data, this strategy accurately detects obstacle positions and trajectories and adjusts path planning in real time. Similarly, in context-aware scenarios, multimodal data fusion significantly improves robots’ adaptability to unknown areas, providing comprehensive solutions for navigation in complex dynamic environments.

### 3.3. Navigation Techniques for Different Task Scenarios

In dynamic environments, the application scenarios of mobile robot navigation are diverse, ranging from indoor navigation to dynamic obstacle avoidance and multi-robot collaboration. Each scenario has distinct requirements for navigation techniques, reflecting the complexity of the environment, the uncertainty of targets, and the unique demands of the task on algorithm adaptability and performance. In recent years, the rapid development of DRL has provided robust technical support for these diverse scenarios, enabling robots to achieve efficient and robust navigation in complex environments.

Indoor navigation is a critical domain for robot applications, characterized by dense obstacles, high environmental dynamics, and the need for highly accurate and adaptive path planning. Liang et al. proposed a context-aware DRL framework designed for mapless navigation in unknown indoor areas [87]. By analyzing environmental context in real time, this method significantly enhanced the robot’s adaptability to environmental changes while optimizing path planning efficiency. Furthermore, Chai et al. developed a hierarchical deep learning control framework by combining RNN with DRL [77]. This framework excelled in unstructured environments, rapidly generating efficient motion trajectories and providing strong support for robot navigation in complex indoor scenarios. These studies demonstrate that DRL can achieve high-precision path planning in dynamic environments by deeply integrating perception and decision-making.

Dynamic obstacle avoidance in complex industrial environments poses greater challenges than indoor navigation. These scenarios require navigation algorithms to manage random variations in moving obstacles while ensuring safety and trajectory smoothness during task execution. Samsani et al. proposed a memory-based group-aware DRL method for effective obstacle avoidance in densely populated environments. By modeling crowd behavior patterns, this method demonstrated strong adaptability to dynamic scenarios, providing critical technical support for obstacle avoidance tasks in complex dynamic environments [88]. These studies highlight DRL’s potential in dynamic environments, particularly for dynamic obstacle avoidance tasks requiring real-time decision-making.

Multi-robot collaboration represents another important application scenario, where complexity arises from the need for coordinated optimization of task allocation and path planning. Unlike single-robot navigation, multi-robot systems must address challenges such as communication delays, path conflicts, and task prioritization. Xu et al. developed a multi-robot collaboration framework based on an improved TD3 algorithm, which significantly enhanced task completion efficiency through multi-step temporal averaging and multi-task optimization [89]. By coordinating information sharing and path planning among multiple robots, this method achieved efficient collaboration in complex environments. The framework also demonstrated excellent performance in task scheduling and conflict resolution, offering new directions for navigation technologies in multi-robot scenarios.

While navigation techniques in different task scenarios have distinct characteristics, they share common challenges in complex dynamic environments, such as the efficiency of real-time decision-making, the ability to integrate multimodal perception, and the precise adaptation to task objectives. In recent years, the application of DRL has not only significantly improved navigation performance in various task scenarios but also suggested directions for future research. By further optimizing DRL algorithm structures and integrating multimodal perception with real-time optimization technologies, robotic navigation systems are expected to demonstrate greater potential in more complex task scenarios.

### 3.4. Analysis of DRL Research Applications

**Figure 3:**
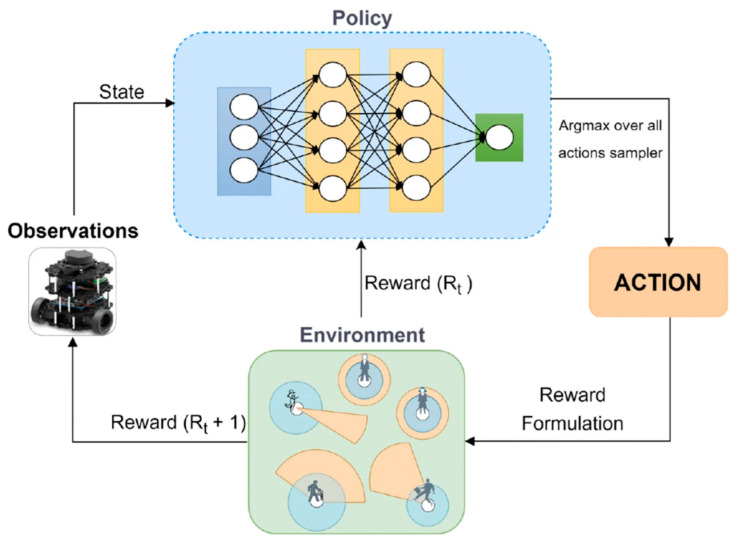
An overview of the deep reinforcement learning framework [90].

In DRL, the agent interacts with the environment by observing states, selecting actions, and receiving rewards, as illustrated in Figure 3. At each time step t, the agent observes the current state, processes it through a policy network to select an action, and receives a scalar reward $r_{t}$ from the environment based on the resulting outcome. The cumulative return over an episode, defined as $R=\sum_{t=0}^{T}\gamma^{t}r_{t}$, where $\gamma \in \left[0,1\right]$ is the discount factor and T is the terminal time step, serves as the core learning signal in DRL. This formulation underpins performance metrics such as max. reward and avg. reward, reported in Table 5. An episode consists of a sequence of such steps and typically terminates when a certain condition is met, such as reaching the goal, exceeding time limits, or encountering a collision with obstacles. Understanding the relationship between stepwise interactions and episode-level evaluations is essential for interpreting training times and policy effectiveness across various navigation scenarios.

**Table 5:** Simple comparison of relevant references.

Application Scenario	Ref.	Algorithm	Perception Type	Training Times	Success Rate	Max. Reward	Avg. Reward	Real System
Indoor	[79]	DWA-RL	L	—	54%	—	—	Y
	[89]	LND3QN	V	1500 episodes	—	100	82	Y
	[91]	A2C	L	—	85%	—	—	Y
	[82]	A New Reward Function	V	—	—	—	—	N
	[83]	GA3C	L	6,000,000 episodes	—	3,000,000	About 2,500,000	N
	[92]	DQN and DDQN	L, V	800 episodes	—	1500	1000	Y
	[10]	DDPG and DQN	—	800,000 steps	—	0	About 0	N
	[93]	DS-DSAC	L	200,000 steps	—	20	About 19	Y
	[94]	GD-RL	L	—	60%	—	—	Y
	[95]	DQN + PTZ	V	10,000 episodes	—	—	—	N
	[87]	Context-Aware DRL Policy	L	2000 steps	98%	—	—	Y
	[96]	β-Decay TL	L	2500 episodes	100%	—	—	N
	[86]	DDPG + PER	V	80,000 steps	—	10	About 8	Y
	[97]	Improved TD3	L	1200 episodes	92.8%	—	—	N
Outdoor	[80]	HGAT-DRL	—	—	90%	—	—	N
	[98]	TERP	V	—	82%	—	—	Y
	[99]	re-DQN	V	—	—	—	—	Y
	[89]	TCAMD	—	2000 episodes	—	About 49	50	N
	[100]	RL-Based MIPP	—	20,000 episodes	—	About 29	30	N
Other	[101]	DDPG	—	800 episodes	—	About 2000	2000	N
	[88]	CAM-RL	—	—	—	—	—	N
	[102]	DODPG	V	600 episodes	—	About −500	0	Y

Note: Success rate—Percentage of episodes in which the agent successfully completed the task. Max. reward—The highest cumulative reward achieved during training or evaluation. Avg. reward—The average cumulative reward across episodes, reflecting the stability and effectiveness of the learned policy. Training times—Represented by either steps (single environment interactions) or episodes (complete navigation trials). Perception type: L = LiDAR-based sensing; V = vision-based sensing. Real system: Y = real robot implementation; N = simulation only. For visual representations of the algorithms summarized in this table, please refer to Appendix A.

In recent years, research on DRL in robot navigation for dynamic environments has developed rapidly. A systematic review of existing studies reveals significant differences and trends in algorithm types, perception modalities, experimental validation methods, task environments, and application scenarios. Table 5 summarizes core information from 22 representative studies published between 2021 and 2024, including algorithm types, perception modalities, validation methods, and scenario distributions, providing data support for analyzing recent research focuses.

Table 5 presents a comparative analysis of various DRL-based navigation approaches in dynamic environments, highlighting differences in application scenarios, algorithm choices, perception modalities, training times, and performance metrics. The data reveal that a significant proportion of studies focus on indoor navigation, where structured environments allow for controlled experiments and reproducible results. In contrast, outdoor navigation studies remain relatively scarce, likely due to the increased complexity of unstructured environments, where variations in terrain, weather conditions, and external disturbances introduce additional challenges for DRL models.

The choice of perception modalities also exhibits a distinct trend, with LiDAR and vision-based sensors being the predominant options. LiDAR is widely employed for its high spatial accuracy and robustness in structured environments, while vision-based approaches offer richer semantic information but suffer from performance fluctuations under dynamic lighting conditions. Studies incorporating multimodal fusion—integrating LiDAR and vision—demonstrate improved navigation robustness, suggesting that a more comprehensive perception strategy contributes to higher success rates in complex environments.

Training durations vary significantly among studies, with some models trained over a few thousand episodes, while others undergo millions of simulation steps. However, longer training durations do not consistently translate into superior performance, indicating that algorithmic efficiency, reward function design, and environmental complexity have a substantial impact on learning outcomes. Furthermore, while several studies report success rates exceeding 90%, many of these evaluations are confined to simulation environments. This discrepancy underscores a critical challenge in DRL research: the gap between simulated performance and real-world deployment.

Real-system validation remains a notable bottleneck, as only half of the studies conduct physical experiments. While simulations offer a cost-effective and scalable means to develop and refine DRL models, real-world testing is essential for assessing adaptability to unpredictable environmental variations. The disparity between high simulation performance and limited real-world implementation suggests that sim-to-real transfer remains an unresolved issue, likely due to domain adaptation challenges, sensor discrepancies, and the unpredictability of real-world interactions.

The findings in Table 5 suggest that while DRL-based navigation has made significant progress in controlled environments, challenges persist in extending these models to practical deployment. The limited number of studies focusing on outdoor navigation, the constraints of single-modality perception, and the ongoing difficulties in real-world validation highlight the need for further advancements in robustness, adaptability, and transferability. The variations in success rates and reward structures across different studies also indicate the influence of task complexity, dataset diversity, and model architecture on performance, reinforcing the necessity of a more standardized evaluation framework for assessing DRL-based navigation in dynamic environments.

## 4. Future Research Directions of Deep Reinforcement Learning in Dynamic Navigation

With the increasing demand for autonomous robot navigation in dynamic and uncertain environments, DRL has shown promising capabilities in handling complex decision-making under real-time constraints. However, its practical deployment still faces significant bottlenecks, including limited policy adaptability to environmental changes, unstable performance during sim-to-real transfer, and difficulty in integrating heterogeneous sensor data for robust perception. These challenges hinder the widespread use of DRL in real-world navigation systems, where failure can lead to safety risks, mission delays, or hardware damage.

To promote reliable and scalable deployment, this section outlines key research directions focused on enhancing navigation-specific perception, adaptive control, and deployment robustness. By addressing these interconnected issues, future DRL systems can achieve greater applicability and safety in complex navigation scenarios. These thematic groups and their corresponding focus areas are summarized in Table 6.

### 4.1. DRL Adaptability and Decision Efficiency in Dynamic Environments

The unpredictability of dynamic environments imposes stringent requirements on the real-time perception and decision-making capabilities of navigation systems. Current DRL algorithms face notable limitations in adapting to real-time dynamic changes, particularly in high-dimensional scenarios. For instance, Politi et al. proposed a 3D navigation strategy for dynamic environments; however, its adaptability to complex, high-dimensional settings remains constrained [103]. Similarly, while Chu et al. and Xiaoyang et al. explore the potential of adaptive reward designs and computationally efficient frameworks, their approaches are not without challenges [104,105], such as balancing reward mechanisms with computational efficiency. Moreover, Wang et al. emphasizes reducing computational complexity as a critical factor for improving real-time performance, but its practical application in resource-constrained embedded systems needs further validation [106].

While these algorithmic enhancements show promise, practical deployment of DRL in real-world navigation systems reveals deeper domain-specific bottlenecks. One critical challenge lies in the high sensitivity of DRL policies to minor changes in environmental dynamics, which often leads to sudden performance degradation in unfamiliar or rapidly evolving scenarios [107,108]. Moreover, safe real-time decision-making is difficult to guarantee, especially under partial observability and delayed feedback, which are common in embedded and outdoor robotic systems. The lack of real-time adaptability not only limits policy robustness but also may cause mission-critical failures in dynamic field conditions [109]. These limitations suggest that enhancing adaptability is a matter not merely of improving learning algorithms but also of addressing uncertainty modeling, online policy correction, and task-specific failure recovery mechanisms [110].

Future research should critically address these limitations. To enhance adaptability, efforts should focus on refining reward mechanisms and improving the efficiency of policy updates. Integrating adaptive reward designs with hierarchical optimization methods may provide robots with better responsiveness to dynamic changes in complex environments. Furthermore, balancing computational efficiency and real-time performance will be pivotal, especially for embedded navigation systems with limited resources [111,112]. In addition, developing lightweight uncertainty-aware policy architectures and hybrid reactive-planning modules can help bridge the gap between learned strategies and safety-critical real-world requirements [110]. By addressing these challenges, DRL-based navigation systems can achieve a significant leap in handling dynamic, high-dimensional environments [109].

### 4.2. Optimizing Multimodal Perception Data Fusion Techniques

Multimodal perception is essential for enhancing navigation robustness in dynamic environments; however, existing data fusion techniques still face significant bottlenecks in real-world deployment. Singh demonstrated the potential of integrating LiDAR, radar, and vision sensors to improve navigation performance [113,114], yet robust methods for modeling heterogeneous sensor data and mitigating cross-modal noise remain underdeveloped. Similarly, while Nissov et al. and Mitta explored radar velocity integration and AI-enhanced fusion [115,116], their solutions often suffer from poor scalability and computational overhead, limiting their applicability in real-time embedded systems. Cheng and Li proposed an open-vocabulary multimodal framework for object detection [117], but its performance under constrained hardware conditions has yet to be validated. Yao et al. emphasized terrain-aware multimodal fusion for robust outdoor navigation [118], but practical applications are still hindered by the trade-off between perception accuracy and computational efficiency.

Beyond algorithmic limitations, real-world deployments of DRL-based navigation systems reveal more fundamental challenges in multimodal perception. First, sensor noise, delays, or asynchronous updates between modalities (e.g., LiDAR vs. camera) can lead to policy divergence or erratic behaviors during navigation [119]. Second, environment-dependent sensor reliability—for example, camera dropout under poor lighting or LiDAR signal occlusion—requires dynamic fusion mechanisms that most current DRL models lack [120]. Third, training-time modality combinations are often inconsistent with test-time availability, causing robustness failures in outdoor or highly dynamic settings [121].

Future research should focus on leveraging graph neural networks and transformer-based architectures for both static and temporal alignment of sensor data, improving generalization under heterogeneous modalities [122]. Additionally, uncertainty-aware fusion models and lightweight cross-modal attention mechanisms may help mitigate runtime conflicts in low-resource embedded platforms [123]. Finally, designing adaptive modality prioritization frameworks, which can selectively ignore unreliable sensors in real time, will be critical to ensuring policy robustness and safety during practical deployment [124,125]. By addressing these multimodal-specific bottlenecks, DRL-based navigation systems can better meet the precision, stability, and safety demands of real-world dynamic environments.

### 4.3. Developing Multi-Robot Collaborative Learning Frameworks

While significant progress has been achieved in single-robot navigation, the collaborative capabilities of multi-robot systems are increasingly vital for improving navigation robustness, efficiency, and scalability in real-world deployments. Raettig identified task allocation and conflict resolution as fundamental challenges in multi-robot collaboration [126]. However, existing collaborative DRL frameworks often exhibit limited scalability, poor communication efficiency, and fragile policy synchronization, especially under high agent density or real-time constraints. Gao et al. and Chen proposed bi-level and swarm-based coordination techniques [127,128], yet their methods struggle with communication delays, policy divergence, and lack of robustness under high system loads. Similarly, Koradiya’s DRL-based resource allocation strategy lacks adaptability to dynamic and unpredictable task demands [129], while Domingo’s task prioritization framework faces trade-offs between execution reliability and computational complexity [130]. Moreover, Dong et al. explored attention-based models for collaborative planning, but their performance degrades in asynchronous multi-agent systems, where input latency and perception misalignment are common [131].

Beyond algorithmic contributions, real-world deployment of multi-robot DRL systems highlights several critical bottlenecks. First, policy inconsistency across agents due to communication dropout or heterogeneous observations can lead to unsafe or suboptimal behaviors in coordinated tasks [132]. Second, non-stationarity of the environment—caused by the mutual influence of agents’ actions—destabilizes learning and undermines long-term convergence [133]. Third, the lack of scalable credit assignment mechanisms hinders the reinforcement signal attribution in large-scale teams, weakening training efficiency and generalization [134,135]. These issues demand DRL frameworks that are not only intelligent but also robust to imperfect communication, task uncertainty, and real-time disturbances.

Future research should prioritize the development of scalable distributed learning architectures and latency-resilient communication protocols that preserve policy coherence across agents in real time [136]. Efforts should also focus on multi-agent temporal abstraction techniques, allowing robots to coordinate at different decision timescales [137]. Finally, integrating predictive task modeling, cross-agent experience sharing, and decentralized reward shaping could significantly enhance the responsiveness and resilience of multi-robot DRL systems in dynamic task environments [138].

### 4.4. Facilitating Sim-to-Real Transfer and Deployment

Simulation environments serve as a cost-effective platform for developing and validating DRL algorithms; however, the transferability of policies from simulation to real-world deployments remains a persistent and critical bottleneck. While Sharma validated DRL’s efficacy in dynamic real-world navigation tasks [50], their method suffered from poor domain adaptation, resulting in inconsistent performance post-transfer. Similarly, Gomez and Muvva et al. emphasized the value of high-fidelity simulations to reduce the sim-to-real performance gap [139,140], but such fidelity often incurs high computational costs and poor scalability. Jeong et al. attempted to improve transferability via distributed learning techniques [141], yet their generalization capability across diverse environments was limited. Wang et al. tackled hardware-level adaptability [142], but balancing accuracy and computational efficiency in resource-constrained robotic platforms remains unresolved. Furthermore, Dong et al. introduced generalized transfer learning frameworks [131], though their robustness in unstructured, highly dynamic field scenarios still demands further evaluation.

Beyond algorithmic limitations, sim-to-real transfer in DRL-based navigation reveals deep-seated challenges unique to reinforcement learning deployment. First, policy overfitting to simulation-specific artifacts—such as ideal sensor noise models or static object dynamics—causes performance degradation in the presence of real-world stochasticity [143,144]. Second, sensor-model mismatch (e.g., between simulated and physical LiDAR) leads to state estimation errors that severely disrupt learned policies [145,146]. Third, training-environment reward structures often fail to capture critical safety and latency constraints that emerge during deployment, leading to brittle behaviors in field scenarios [147,148]. Finally, most DRL models assume perfect observability and stationary dynamics—conditions rarely met in practice—resulting in failures to adapt during execution [149].

Future research should therefore focus on developing lightweight, real-time-capable simulation environments that retain task-relevant dynamics while remaining computationally efficient [150]. Techniques such as domain randomization, adaptive simulation calibration, and adversarial sim-to-real augmentation should be refined to improve robustness against domain shift [151,152]. Additionally, simulation-aware policy architectures, including feature disentanglement and latent-space alignment, may offer improved generalization [153]. Optimizing DRL agents for cross-platform compatibility and integrating online policy correction mechanisms post-deployment will be crucial for closing the sim-to-real loop [154]. Ultimately, sim-to-real transfer must evolve from an offline pre-training pipeline to an online adaptive paradigm that dynamically aligns learned policies with real-world uncertainty and perception variability [155].

### 4.5. Strengthening Safety and Explainability

Navigation tasks in dynamic environments often involve high-risk scenarios, where a single decision error can result in mission failure or physical harm. In such contexts, ensuring both safety and explainability of DRL algorithms becomes a critical prerequisite for real-world deployment. Malaiyappan and Sistla emphasized the need for robust decision-making frameworks in complex navigation tasks [156]. However, most current DRL algorithms operate as black-box systems, offering limited insight into their internal reasoning and failing to guarantee safe behavior under uncertainty. Although Szolc et al. proposed using tangled program graphs to enhance model transparency [157], these solutions often sacrifice real-time responsiveness and computational tractability—two key factors in time-critical navigation tasks.

Moreover, explainability is not merely an academic requirement, but a practical necessity for verifying DRL decisions in safety-critical applications. Gao and Li advocated for interpretable strategy generation [127], yet systematic frameworks to quantify or audit such interpretability remain immature. Wang also emphasized the role of robust perception in safety assurance [98], but integrating perception-driven safety constraints into DRL policy learning remains a challenge—particularly under computation and latency constraints of embedded systems.

Beyond theoretical approaches, deployment experiences have revealed deeper reinforcement-learning-specific safety bottlenecks. First, DRL agents often learn through trial-and-error, making them prone to unsafe exploration behaviors unless explicitly constrained [158]. Second, the lack of interpretable failure modes makes debugging and certification difficult in critical systems [159,160]. Third, reward hacking, where agents optimize unintended behaviors due to misaligned rewards, poses a severe safety threat in unpredictable environments [161]. These issues collectively hinder the ethical and dependable use of DRL in real-world robotic systems.

Future research should prioritize integrating adaptive safety shields and online risk-aware learning modules within DRL frameworks to proactively mitigate unsafe actions during execution [162]. Additionally, combining DRL with symbolic logic, causal models, or model-checking-based verification may enhance policy transparency while preserving learning flexibility [163]. Finally, efforts should be made to establish benchmark datasets and evaluation protocols for DRL interpretability and safety, promoting reproducibility and trustworthy deployment [164]. By addressing these domain-specific gaps, future systems can achieve robust, safe, and accountable navigation in dynamic and high-risk environments.

### 4.6. Cross-Domain Applications and Extensions

As DRL technology matures, its application scope is expanding into real-world domains such as industrial automation, intelligent transportation, and medical assistance [14,24]. However, these domains impose unique navigation-related constraints, where policy failures may lead to serious operational or safety consequences. In industrial scenarios, mobile robots must perform precise path planning in dense multi-agent environments, where minor trajectory delays or scheduling misalignment can cause bottlenecks or collisions [3]. In medical assistance scenarios, the navigation system must operate in crowded and dynamic environments, requiring not only accurate obstacle avoidance but also strict adherence to safety margins around humans [165]. These domain-specific demands call for DRL algorithms to move beyond academic benchmarks and confront real-world deployment complexity. Three foundational research directions—multimodal perception, real-time adaptability, and sim-to-real transfer—form an interdependent triad supporting reliable navigation.

First, multimodal perception technologies integrate diverse information from LiDAR [166], vision, and other sensors, enhancing spatial understanding in cluttered environments. However, sensor desynchronization and modality conflict can produce erroneous state estimates, leading to oscillatory motion, incorrect turning, or failure to detect dynamic obstacles [167].

Second, real-time adaptability is essential to allow DRL agents to adjust trajectories in response to sudden events such as pedestrian intrusion, road blockage, and layout change. Yet, in many cases, DRL policies suffer from slow reaction under uncertainty, especially when deployed without sufficient online fine-tuning.

Third, sim-to-real transfer remains a critical bottleneck. Training on idealized simulation data often leads to policy overfitting, and when deployed in real-world scenarios with sensor noise or actuator delay, these agents may exhibit unstable paths, reward misalignment, or catastrophic navigation failures [168,169].

Moreover, these three areas are mutually reinforcing. Better multimodal perception supports more realistic simulation inputs, improving sim-to-real generalization [168]. Enhanced sim-to-real robustness, in turn, demands real-time adaptability to handle variations not seen during training [169]. Real-time adaptability depends on reliable, low-latency perception fusion, creating a feedback loop among these components [170]. The failure of one capability can propagate to others, ultimately causing task-level breakdowns in deployed DRL navigation systems.

Future research should focus on developing navigation-specific DRL strategies that incorporate sensor-aware policy adjustment, uncertainty-guided trajectory re-planning, and fallback decision mechanisms in case of perception or adaptation failure [143,153]. Domain-specific deployments would also benefit from cross-modal consistency models, reward alignment checks, and continual simulation correction modules [122,152]. By resolving these interconnected navigation risks, DRL can be more reliably deployed in complex, dynamic, and high-stakes real-world environments.

In essence, multimodal perception, real-time adaptability, and sim-to-real transfer are not isolated research challenges but rather mutually reinforcing pillars. Addressing one often facilitates progress in the others, forming a virtuous cycle critical for robust DRL navigation in real-world systems [155].

## 5. Conclusions

This paper systematically explores the theoretical foundations, classical algorithms, and key technologies of DRL in dynamic environment navigation. Through a comparative analysis of classical algorithms such as DQN, PPO, and SAC, the study reveals the strengths and limitations of different methods in terms of adaptability, robustness, and computational efficiency. Furthermore, the paper provides an in-depth analysis of the technical advancements of DRL in addressing core challenges of dynamic environments, including adaptability, multimodal perception data fusion, and multi-robot collaboration.

The findings indicate that DRL, by combining the feature extraction capabilities of deep learning with the decision-making optimization of reinforcement learning, offers efficient and flexible solutions for navigation systems in dynamic environments. Nonetheless, existing technologies still face significant challenges in handling high-dimensional dynamic environments, achieving sim-to-real transfer, and ensuring system safety and interpretability.

Looking ahead, with the advancement of technology and increasing demands for autonomous systems, the potential of DRL in dynamic navigation will continue to expand. To support reliable deployment, future research should focus on breakthroughs in multimodal data fusion, collaborative learning frameworks, sim-to-real transfer, and algorithm safety and interpretability. These efforts will not only drive theoretical progress, but also lay the foundation for DRL’s practical application in intelligent robotics, industrial automation, and smart transportation.

## Figures and Tables

**Table 1:** Recent review of deep reinforcement learning in mobile robot navigation.

Ref.	Key Findings
[21]	This study categorizes path planning methodologies into four main types: learning-based, space-based, time-based, and environment-based approaches. It introduces a novel taxonomy that encompasses the transition from classical to state-of-the-art methods for path planning in dynamic environments.
[22]	This systematic review explores the application of DRL in mobile robot navigation within hazardous environments. It classifies navigation approaches into three categories—autonomous-based, SLAM-based, and planning-based navigation—and analyzes their respective strengths and weaknesses.
[23]	This review examines the applications, advantages, and limitations of deep learning in robotic systems, providing an analysis based on contemporary research.
[4]	This paper reviews AI-enhanced navigation strategies for mobile robots, highlighting the distinctions among different approaches.
[24]	This study systematically introduces and summarizes existing DRL-based exploration methods and discusses their potential applications in robot attitude control tasks.
[25]	This paper provides an overview of fundamental concepts in deep reinforcement learning, including value functions and policy gradient algorithms, and discusses their applications in mobile robot path planning.
[26]	This review investigates DRL methods and DRL-based navigation frameworks, systematically comparing and analyzing the similarities and differences in four typical application scenarios.

**Table 2:** Comparison of robot navigation strategies.

**Ref.**	**Method**	**Advantages**	**Disadvantages**	**Best Suited for**
[41,42,43]	Classical Control (Dijkstra, A*, RRT, DWA)	Deterministic, mathematically well-founded, efficient in structured/static environments	Poor adaptability in dynamic settings, requires frequent re-planning	Structured and static environments with predefined obstacles
[13]	Deep Reinforcement Learning (DRL)	Adaptive, real-time decision-making, good generalization in non-stationary settings	High training complexity, requires extensive computational resources and well-designed reward functions	Dynamic and unpredictable environments requiring flexible navigation
[44,45]	Hybrid Learning (Neuro-SLAM, Imitation Learning)	Improves perception and localization (Neuro-SLAM), faster learning from expert demonstrations (imitation learning)	Limited adaptability due to reliance on pre-trained models (Neuro-SLAM), requires high-quality expert data (imitation learning)	Enhancing classical navigation methods with learning-based adaptations
[46,47]	Multi-Agent Reinforcement Learning (MARL)	Enables cooperation, adaptive to teammates/adversaries, efficient for multi-robot tasks	Communication constraints, higher computational complexity, policy convergence challenges	Collaborative multi-robot navigation and swarm intelligence

**Table 6:** Thematic grouping of DRL deployment challenges.

Thematic Group	Included Sections	Focus Area
Adaptability and Perception	Section 4.1 and Section 4.2	Real-time policy adaptation and multimodal sensory integration
Collaboration and Transfer	Section 4.3, Section 4.4 and Section 4.5	Sim-to-real knowledge transfer and multi-robot coordination
Safety and Deployment Robustness	Section 4.4, Section 4.5 and Section 4.6	Interpretability, algorithmic safety, and cross-domain generalization

## Data Availability

No new data were generated or analyzed in this study. Data sharing is not applicable to this article.

## Abbreviations

The following abbreviations are used in this manuscript.

DRL	Deep reinforcement learning
SLAM	Simultaneous localization and mapping
RRT	Rapidly exploring random tree
MDPs	Markov decision processes
RL	Reinforcement learning
DNNs	Deep neural networks
DQN	Deep Q-network
DDPG	Deep deterministic policy gradient
TD3	Twin delayed deep deterministic policy gradient
PPO	Proximal policy optimization
SAC	Soft actor-critic
DWA	Dynamic window approach
MARL	Multi-agent reinforcement learning
Double DQN	Double deep Q-network
Dueling DQN	Dueling deep Q-network
TRPO	Trust region policy optimization
A3C	Asynchronous advantage actor-critic
KL divergence	Kullback–Leibler divergence
A*	A-Star

## Appendix A

This appendix presents structural frameworks and flowcharts of several representative DRL-based navigation methods discussed in the review. These visualizations are provided to help readers better understand the architectural design and information flow of the respective approaches.

**Table A1:** Summary of structural frameworks and flowcharts of selected DRL-based navigation methods.

Ref.	Method	Framework/Flowchart
[33,58]	Deep Q-Network (DQN)	
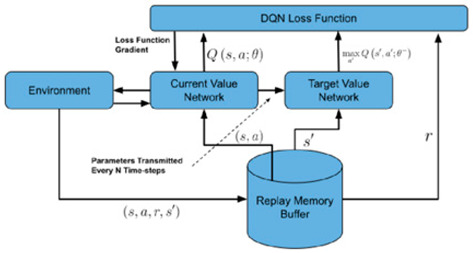
[57,60]	Double Deep Q-Network (Double DQN)	
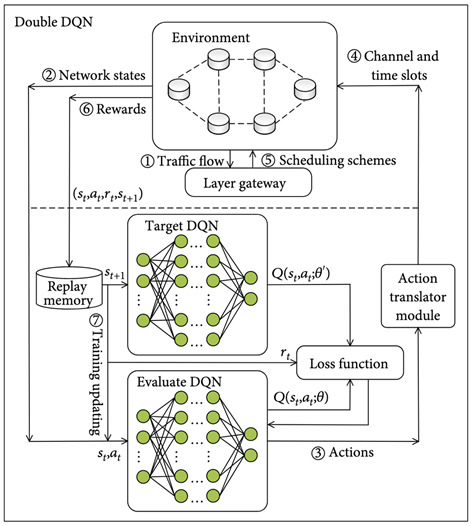
[61,65]	Dueling Deep Q-Network (Dueling DQN)	
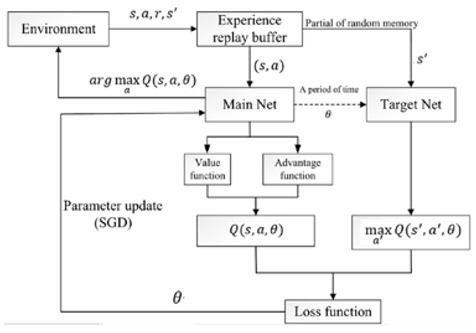
[8,37]	Trust Region Policy Optimization (TRPO)	
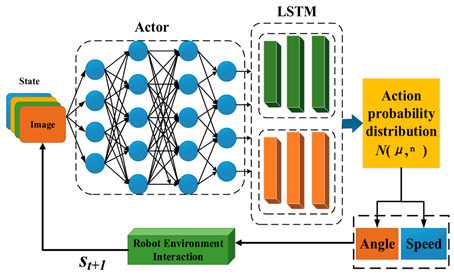
[37,66]	Proximal Policy Optimization (PPO)	
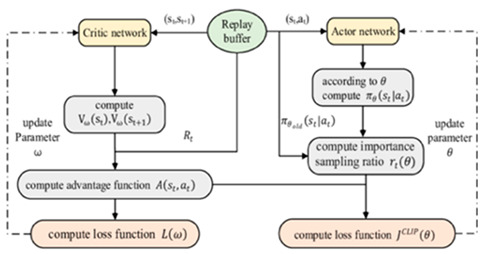
[67,68]	Asynchronous Advantage Actor-Critic (A3C)	
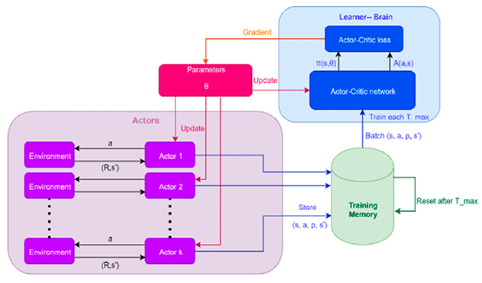
[16,69]	Deep Deterministic Policy Gradient (DDPG)	
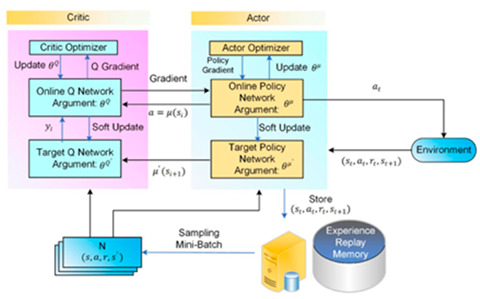
[36,70]	Soft Actor-Critic (SAC)	
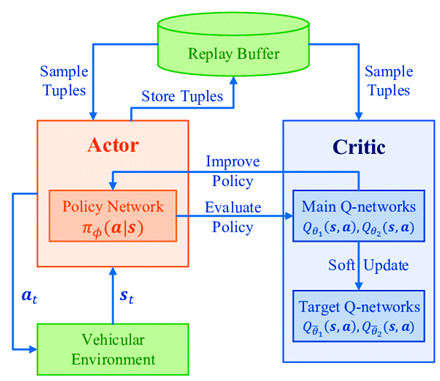
[35,71]	Twin Delayed Deep Deterministic Policy Gradient (TD3)	
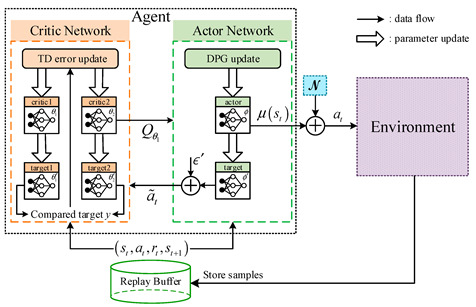
[79]	DWA-RL	
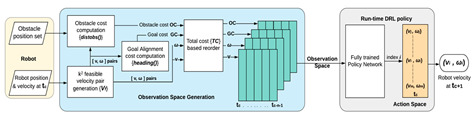
[89]	LND3QN	
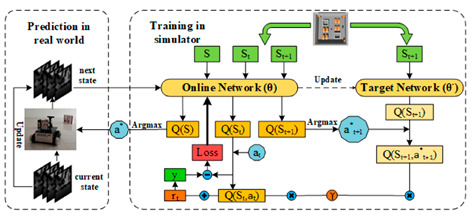
[91]	A2C	
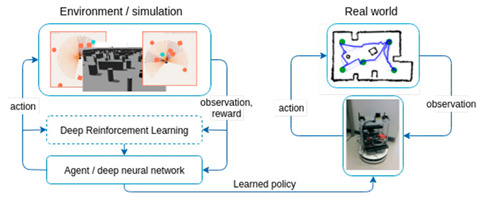
[82]	A New Reward Function	
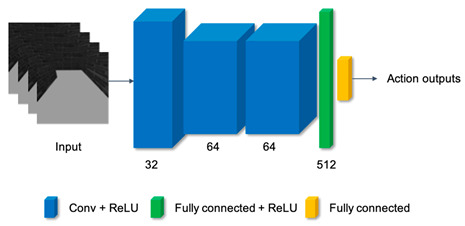
[83]	GA3C	
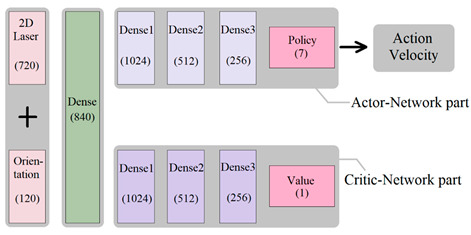
[92]	DQN and DDQN	
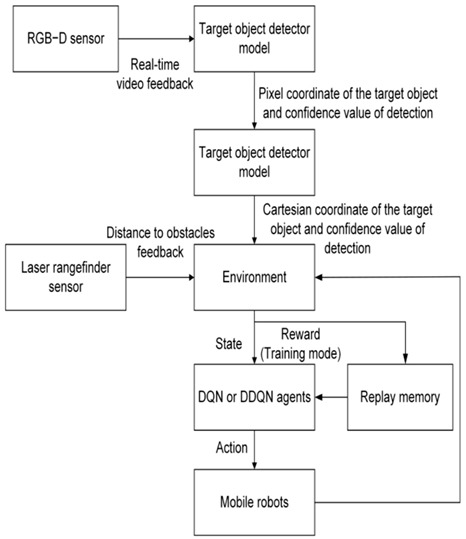
[10]	DDPG and DQN	
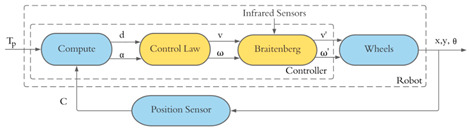
[93]	DS-DSAC	
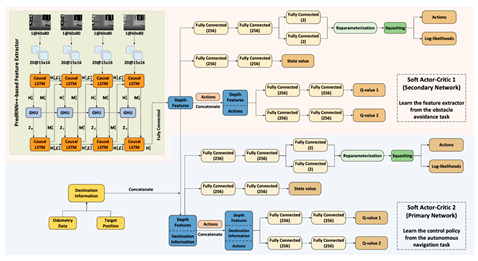
[94]	GD-RL	
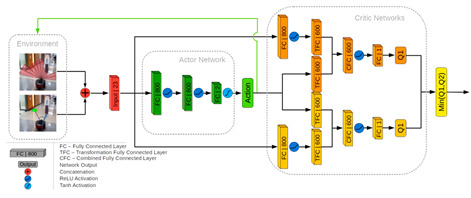
[95]	DQN + PTZ	
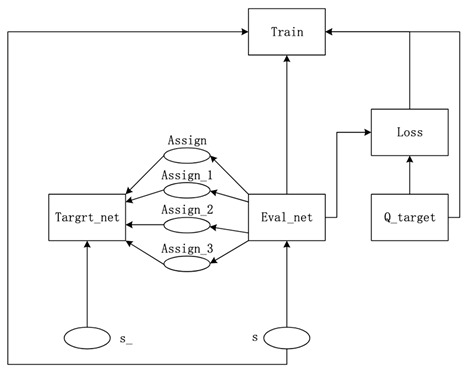
[87]	Context-Aware DRL Policy	
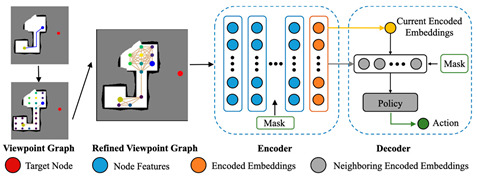
[96]	β-Decay TL	
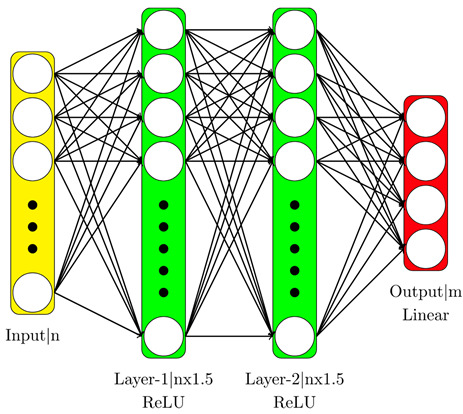
[86]	DDPG + PER	
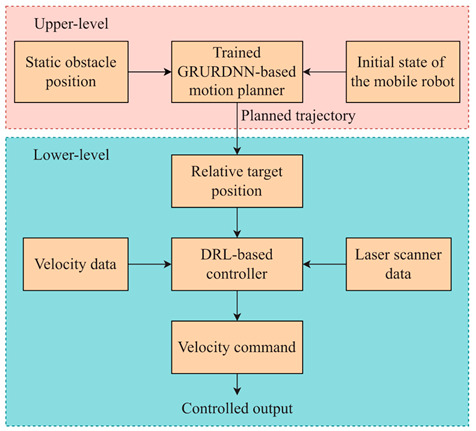
[97]	Improved TD3	
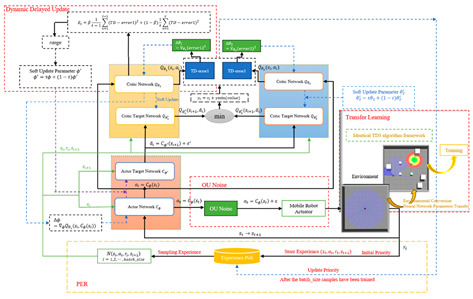
[80]	HGAT-DRL	
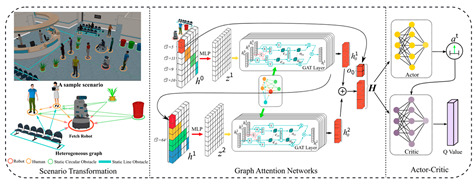
[98]	TERP	
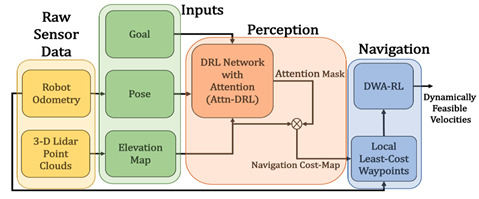
[99]	re-DQN	
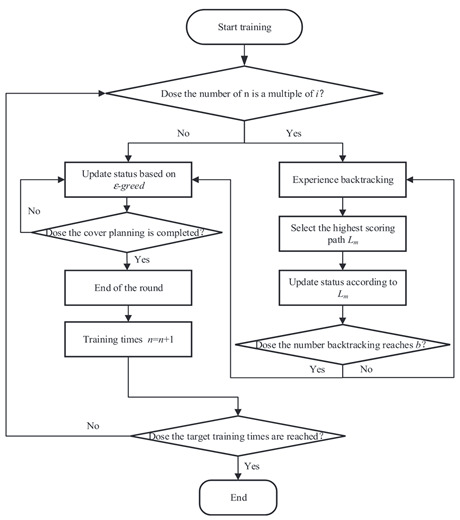
[89]	TCAMD	
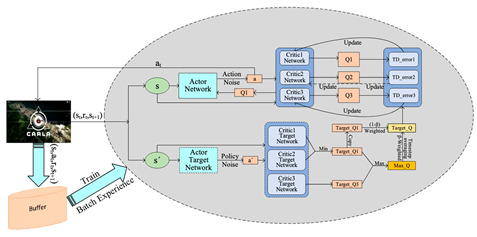
[100]	RL-Based MIPP	
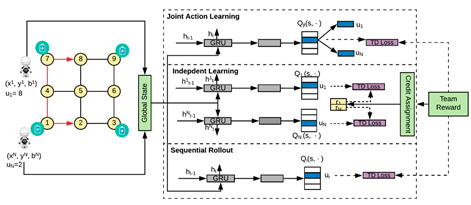
[79]	CAM-RL	
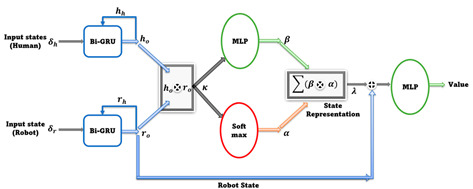
[92]	DODPG	

## References

[1] Prasuna R.G., Potturu S.R. (2024). Deep Reinforcement Learning in Mobile Robotics—A Concise Review. Multimed. Tools Appl..

[2] Siciliano B., Khatib O., Siciliano B., Khatib O. (2016). Robotics and the Handbook. Springer Handbook of Robotics.

[3] Chen L., Jiang Z., Cheng L., Knoll A.C., Zhou M. (2022). Deep Reinforcement Learning Based Trajectory Planning Under Uncertain Constraints. Front. Neurorobot..

[4] Nasti S.M., Chishti M.A. A Review of AI-Enhanced Navigation Strategies for Mobile Robots in Dynamic Environments. Proceedings of the 2024 ASU International Conference in Emerging Technologies for Sustainability and Intelligent Systems (ICETSIS).

[5] Duan C., Junginger S., Huang J., Jin K., Thurow K. (2019). Deep Learning for Visual SLAM in Transportation Robotics: A Review. Transp. Saf. Environ..

[6] LaValle S.M. (2006). Planning Algorithms.

[7] Bhagat S., Banerjee H., Tse Z.T.H., Ren H. (2019). Deep Reinforcement Learning for Soft, Flexible Robots: Brief Review with Impending Challenges. Robotics.

[8] Sun H., Zhang W., Yu R., Zhang Y. (2021). Motion Planning for Mobile Robots—Focusing on Deep Reinforcement Learning: A Systematic Review. IEEE Access.

[9] Devo A., Mezzetti G., Costante G., Fravolini M.L., Valigi P. (2020). Towards Generalization in Target-Driven Visual Navigation by Using Deep Reinforcement Learning. IEEE Trans. Robot..

[10] Quiroga F., Hermosilla G., Farias G., Fabregas E., Montenegro G. (2022). Position Control of a Mobile Robot through Deep Reinforcement Learning. Appl. Sci..

[11] Sangiovanni B., Incremona G.P., Piastra M., Ferrara A. (2021). Self-Configuring Robot Path Planning With Obstacle Avoidance via Deep Reinforcement Learning. IEEE Control Syst. Lett..

[12] Candra A., Budiman M.A., Hartanto K. Dijkstra’s and A-Star in Finding the Shortest Path: A Tutorial. Proceedings of the 2020 International Conference on Data Science, Artificial Intelligence, and Business Analytics (DATABIA).

[13] Mnih V., Kavukcuoglu K., Silver D., Rusu A.A., Veness J., Bellemare M.G., Graves A., Riedmiller M., Fidjeland A.K., Ostrovski G. (2015). Human-Level Control through Deep Reinforcement Learning. Nature.

[14] Kober J., Bagnell J.A., Peters J. (2013). Reinforcement Learning in Robotics: A Survey. Int. J. Robot. Res..

[15] Zhang H., Liu L.Z., Xie H., Jiang Y., Zhou J., Wang Y. (2022). Deep Learning-Based Robot Vision: High-End Tools for Smart Manufacturing. IEEE Instrum. Meas. Mag..

[16] Lillicrap T.P., Hunt J.J., Pritzel A., Heess N., Erez T., Tassa Y., Silver D., Wierstra D. (2019). Continuous Control with Deep Reinforcement Learning. arXiv.

[17] Miranda V.R.F., Neto A.A., Freitas G.M., Mozelli L.A. (2024). Generalization in Deep Reinforcement Learning for Robotic Navigation by Reward Shaping. IEEE Trans. Ind. Electron..

[18] Sutton R.S., Barto A.G. (2018). Reinforcement Learning.

[19] Nguyen H., La H. Review of Deep Reinforcement Learning for Robot Manipulation. Proceedings of the 2019 Third IEEE International Conference on Robotic Computing (IRC).

[20] Garaffa L.C., Basso M., Konzen A.A., De Freitas E.P. (2023). Reinforcement Learning for Mobile Robotics Exploration: A Survey. IEEE Trans. Neural Netw. Learn. Syst..

[21] Sharma G., Jain S., Sharma R.S. (2025). Path Planning for Fully Autonomous UAVs-a Taxonomic Review and Future Perspectives. IEEE Access.

[22] Le H., Saeedvand S., Hsu C.-C. (2024). A Comprehensive Review of Mobile Robot Navigation Using Deep Reinforcement Learning Algorithms in Crowded Environments. J. Intell. Robot. Syst..

[23] Pierson H.A., Gashler M.S. (2017). Deep Learning in Robotics: A Review of Recent Research. Adv. Robot..

[24] Li C., Wu F., Zhao J., Sun F., Cangelosi A., Zhang J., Yu Y., Liu H., Fang B. (2023). A Review of Deep Reinforcement Learning Exploration Methods: Prospects and Challenges for Application to Robot Attitude Control Tasks. Cognitive Systems and Information Processing, Proceedings of the 7th International Conference, ICCSIP 2022, Fuzhou, China, 17–18 December 2022.

[25] Zhao Y., Zhang Y., Wang S. (2021). A Review of Mobile Robot Path Planning Based on Deep Reinforcement Learning Algorithm. J. Phys. Conf. Ser..

[26] Zhu K., Zhang T. (2021). Deep Reinforcement Learning Based Mobile Robot Navigation: A Review. Tsinghua Sci. Technol..

[27] Hu Y., Ye D., Kang J., Wu M., Yu R. (2024). A Cloud-Edge Collaborative Architecture for Multimodal LLMs-Based Advanced Driver Assistance Systems in IoT Networks. IEEE Internet Things J..

[28] Jiang H., Wang H., Yau W.-Y., Wan K.-W. A Brief Survey: Deep Reinforcement Learning in Mobile Robot Navigation. Proceedings of the 2020 15th IEEE Conference on Industrial Electronics and Applications (ICIEA).

[29] Schulman J., Wolski F., Dhariwal P., Radford A., Klimov O. (2017). Proximal Policy Optimization Algorithms. arXiv.

[30] Bellman R. (1957). A Markovian Decision Process. J. Math. Mech..

[31] Mnih V., Kavukcuoglu K., Silver D., Graves A., Antonoglou I., Wierstra D., Riedmiller M. (2013). Playing Atari with Deep Reinforcement Learning. arXiv.

[32] Silver D., Huang A., Maddison C.J., Guez A., Sifre L., Van Den Driessche G., Schrittwieser J., Antonoglou I., Panneershelvam V., Lanctot M. (2016). Mastering the Game of Go with Deep Neural Networks and Tree Search. Nature.

[33] Mnih V., Badia A.P., Mirza M., Graves A., Lillicrap T.P., Harley T., Silver D., Kavukcuoglu K. (2016). Asynchronous Methods for Deep Reinforcement Learning. arXiv.

[34] Qian T., Wang M. Optimal Ancillary Service Disaggregation for EV Charging Station Aggregators: A Hybrid On–Off Policy Reinforcement Learning Framework, 2024. https://papers.ssrn.com/sol3/papers.cfm?abstract_id=5100232.

[35] Fujimoto S., van Hoof H., Meger D. (2018). Addressing Function Approximation Error in Actor-Critic Methods. arXiv.

[36] Haarnoja T., Zhou A., Abbeel P., Levine S. (2018). Soft Actor-Critic: Off-Policy Maximum Entropy Deep Reinforcement Learning with a Stochastic Actor. arXiv.

[37] Schulman J., Levine S., Moritz P., Jordan M.I., Abbeel P. (2017). Trust Region Policy Optimization. arXiv.

[38] Nam S., Nguyen T.A., Choi E., Min D. (2025). Multi-Head Fusion-Based Actor-Critic Deep Reinforcement Learning with Memory Contextualisation for End-to-End Autonomous Navigation. TechRxiv.

[39] Yang G., Guo Y. Deep Reinforcement Learning Based Mobile Robot Navigation in Crowd Environments. Proceedings of the 2024 21st International Conference on Ubiquitous Robots (UR).

[40] Parooei M., Tale Masouleh M., Kalhor A. (2024). MAP3F: A Decentralized Approach to Multi-Agent Pathfinding and Collision Avoidance with Scalable 1D, 2D, and 3D Feature Fusion. Intell. Serv. Robot..

[41] Dijkstra E.W. (2022). A Note on Two Problems in Connexion with Graphs. Edsger Wybe Dijkstra: His Life, Work, and Legacy.

[42] Hart P.E., Nilsson N.J., Raphael B. (1968). A Formal Basis for the Heuristic Determination of Minimum Cost Paths. IEEE Trans. Syst. Sci. Cybern..

[43] Fox D., Burgard W., Thrun S. (1997). The Dynamic Window Approach to Collision Avoidance. IEEE Robot. Autom. Mag..

[44] Gupta S., Tolani V., Davidson J., Levine S., Sukthankar R., Malik J. (2019). Cognitive Mapping and Planning for Visual Navigation. arXiv.

[45] Cadena C., Carlone L., Carrillo H., Latif Y., Scaramuzza D., Neira J., Reid I., Leonard J.J. (2016). Past, Present, and Future of Simultaneous Localization and Mapping: Toward the Robust-Perception Age. IEEE Trans. Robot..

[46] Chen J., Ma R., Oyekan J. (2023). A Deep Multi-Agent Reinforcement Learning Framework for Autonomous Aerial Navigation to Grasping Points on Loads. Robot. Auton. Syst..

[47] Yu C., Yang X., Gao J., Yang H., Wang Y., Wu Y., Avidan S., Brostow G., Cissé M., Farinella G.M., Hassner T. (2022). Learning Efficient Multi-Agent Cooperative Visual Exploration. Computer Vision—ECCV 2022.

[48] Sarfi M.H. (2024). Autonomous Exploration and Mapping of Unknown Environments. Master’s Thesis.

[49] Ahmed S., Azar A.T., Sardar M.Z., Haider Z., Kamal N.A. (2024). Exploring Reinforcement Learning Techniques in the Realm of Mobile Robotics. IJAAC.

[50] Sharma R. (2024). Optimizing Deep Reinforcement Learning for Real-World Robotics: Challenges and Solutions. Int. J. Artif. Intell. Comput. Sci. Manag. Technol..

[51] Tsiotras P., Gombolay M., Foerster J. (2024). Editorial: Decision-Making and Planning for Multi-Agent Systems. Front. Robot. AI.

[52] Karwowski J., Szynkiewicz W. Human-Aware Robot Trajectory Planning with Hybrid Candidate Generation: Leveraging a Pedestrian Motion Model for Diverse Trajectories. Proceedings of the 2024 13th International Workshop on Robot Motion and Control (RoMoCo).

[53] Vaidya H., Dhabliya D., Jweeg M., Almusawi M., Naser Z.L., Hashem A., Jawad A.Q. An Empirical Analysis of Various Techniques of Solving Obstacles through Artificial Intelligence. Proceedings of the 2024 4th International Conference on Advance Computing and Innovative Technologies in Engineering (ICACITE).

[54] Rong S., Meng R., Guo J., Cui P., Qiao Z. (2024). Multi-Vehicle Collaborative Planning Technology under Automatic Driving. Sustainability.

[55] Sorokin M., Tan J., Liu C.K., Ha S. (2021). Learning to Navigate Sidewalks in Outdoor Environments. arXiv.

[56] Li P., An Z., Abrar S., Zhou L. (2025). Large Language Models for Multi-Robot Systems: A Survey. arXiv.

[57] Alsadie D. (2024). A Comprehensive Review of AI Techniques for Resource Management in Fog Computing: Trends, Challenges, and Future Directions. IEEE Access.

[58] Fan T., Long P., Liu W., Pan J. (2020). Distributed Multi-Robot Collision Avoidance via Deep Reinforcement Learning for Navigation in Complex Scenarios. Int. J. Robot. Res..

[59] You K., Zhou C., Ding L. (2023). Deep Learning Technology for Construction Machinery and Robotics. Autom. Constr..

[60] Hasselt H.v., Guez A., Silver D. (2016). Deep Reinforcement Learning with Double Q-Learning. Proc. AAAI Conf. Artif. Intell..

[61] Wang Y., Fang Y., Lou P., Yan J., Liu N. (2020). Deep Reinforcement Learning Based Path Planning for Mobile Robot in Unknown Environment. J. Phys. Conf. Ser..

[62] Haarnoja T., Ha S., Zhou A., Tan J., Tucker G., Levine S. (2019). Learning to Walk via Deep Reinforcement Learning. arXiv.

[63] Deep Reinforcement Learning for Scheduling in an Edge Computing-Based Industrial Internet of Things. https://www.researchgate.net/publication/355377739_Deep_Reinforcement_Learning_for_Scheduling_in_an_Edge_Computing-Based_Industrial_Internet_of_Things.

[64] Hu M., Zhang J., Matkovic L., Liu T., Yang X. (2023). Reinforcement Learning in Medical Image Analysis: Concepts, Applications, Challenges, and Future Directions. J. Appl. Clin. Med. Phys..

[65] Wang Y., Li X., Wan P., Chang L., Deng X. (2022). Dueling Deep Q-Networks for Social Awareness-Aided Spectrum Sharing. Complex Intell. Syst..

[66] Wang L., Feng X., Zhang R., Hou Z., Wang G., Zhang H. (2024). Energy Management of Integrated Energy System in the Park under Multiple Time Scales. AIMS Energy.

[67] Wang Z., Schaul T., Hessel M., Hasselt H.V., Lanctot M., de Freitas N. Dueling Network Architectures for Deep Reinforcement Learning. Proceedings of the 33rd International Conference on Machine Learning (ICML).

[68] Nakabi T.A., Toivanen P. (2021). Deep Reinforcement Learning for Energy Management in a Microgrid with Flexible Demand. Sustain. Energy Grids Netw..

[69] Dong R., Du J., Liu Y., Heidari A.A., Chen H. (2023). An Enhanced Deep Deterministic Policy Gradient Algorithm for Intelligent Control of Robotic Arms. Front. Neuroinform..

[70] Shi J., Du J., Wang J., Wang J., Yuan J. (2020). Priority-Aware Task Offloading in Vehicular Fog Computing Based on Deep Reinforcement Learning. IEEE Trans. Veh. Technol..

[71] Liu S., Yang Z., Zhang Z., Jiang R., Ren T., Jiang Y., Chen S., Zhang X. (2022). Application of Deep Reinforcement Learning in Reconfiguration Control of Aircraft Anti-Skid Braking System. Aerospace.

[72] Arce D., Solano J., Beltrán C. (2023). A Comparison Study between Traditional and Deep-Reinforcement-Learning-Based Algorithms for Indoor Autonomous Navigation in Dynamic Scenarios. Sensors.

[73] Reda D. (2025). Physics-Based Character Controllers with Reinforcement Learning. Ph.D. Thesis.

[74] Cai B., Wei C., Ji Z. (2025). Deep Reinforcement Learning with Multiple Unrelated Rewards for AGV Mapless Navigation. IEEE Trans. Autom. Sci. Eng..

[75] Zhu Z., Lin K., Jain A.K., Zhou J. (2023). Transfer Learning in Deep Reinforcement Learning: A Survey. IEEE Trans. Pattern Anal. Mach. Intell..

[76] Tai L., Paolo G., Liu M. (2017). Virtual-to-Real Deep Reinforcement Learning: Continuous Control of Mobile Robots for Mapless Navigation. arXiv.

[77] Kuwata Y., Teo J., Fiore G., Karaman S., Frazzoli E., How J.P. (2009). Real-Time Motion Planning With Applications to Autonomous Urban Driving. IEEE Trans. Control Syst. Technol..

[78] Cao X., Sun C., Yan M. (2019). Target Search Control of AUV in Underwater Environment With Deep Reinforcement Learning. IEEE Access.

[79] Patel U., Kumar N.K.S., Sathyamoorthy A.J., Manocha D. DWA-RL: Dynamically Feasible Deep Reinforcement Learning Policy for Robot Navigation among Mobile Obstacles. Proceedings of the 2021 IEEE International Conference on Robotics and Automation (ICRA).

[80] Zhou Z., Zeng Z., Lang L., Yao W., Lu H., Zheng Z., Zhou Z. (2022). Navigating Robots in Dynamic Environment With Deep Reinforcement Learning. IEEE Trans. Intell. Transp. Syst..

[81] Feng S., Sebastian B., Ben-Tzvi P. (2021). A Collision Avoidance Method Based on Deep Reinforcement Learning. Robotics.

[82] Wenzel P., Schön T., Leal-Taixé L., Cremers D. Vision-Based Mobile Robotics Obstacle Avoidance With Deep Reinforcement Learning. Proceedings of the 2021 IEEE International Conference on Robotics and Automation (ICRA).

[83] Beomsoo H., Ravankar A.A., Emaru T. Mobile Robot Navigation Based on Deep Reinforcement Learning with 2D-LiDAR Sensor Using Stochastic Approach. Proceedings of the 2021 IEEE International Conference on Intelligence and Safety for Robotics (ISR).

[84] Kaymak Ç., Uçar A., Güzeliş C. (2023). Development of a New Robust Stable Walking Algorithm for a Humanoid Robot Using Deep Reinforcement Learning with Multi-Sensor Data Fusion. Electronics.

[85] An G., Zhang S. (2023). Pruning Replay Buffer for Efficient Training of Deep Reinforcement Learning. J. Emerg. Investig..

[86] Chai R., Niu H., Carrasco J., Arvin F., Yin H., Lennox B. (2024). Design and Experimental Validation of Deep Reinforcement Learning-Based Fast Trajectory Planning and Control for Mobile Robot in Unknown Environment. IEEE Trans. Neural Netw. Learn. Syst..

[87] Liang J., Wang Z., Cao Y., Chiun J., Zhang M., Sartoretti G.A. Context-Aware Deep Reinforcement Learning for Autonomous Robotic Navigation in Unknown Area. Proceedings of the 7th Conference on Robot Learning.

[88] Samsani S.S., Mutahira H., Muhammad M.S. (2023). Memory-Based Crowd-Aware Robot Navigation Using Deep Reinforcement Learning. Complex Intell. Syst..

[89] Xu T., Meng Z., Lu W., Tong Z. (2024). End-to-End Autonomous Driving Decision Method Based on Improved TD3 Algorithm in Complex Scenarios. Sensors.

[90] Montero E.E., Mutahira H., Pico N., Muhammad M.S. (2024). Dynamic Warning Zone and a Short-Distance Goal for Autonomous Robot Navigation Using Deep Reinforcement Learning. Complex Intell. Syst..

[91] Dobrevski M., Skočaj D. (2021). Deep Reinforcement Learning for Map-Less Goal-Driven Robot Navigation. Int. J. Adv. Robot. Syst..

[92] Lee M.-F.R., Yusuf S.H. (2022). Mobile Robot Navigation Using Deep Reinforcement Learning. Processes.

[93] Wu K., Wang H., Esfahani M.A., Yuan S. (2022). Learn to Navigate Autonomously Through Deep Reinforcement Learning. IEEE Trans. Ind. Electron..

[94] Cimurs R., Suh I.H., Lee J.H. (2022). Goal-Driven Autonomous Exploration Through Deep Reinforcement Learning. IEEE Robot. Autom. Lett..

[95] Zheng J., Mao S., Wu Z., Kong P., Qiang H. (2022). Improved Path Planning for Indoor Patrol Robot Based on Deep Reinforcement Learning. Symmetry.

[96] Kumaar A.A.N., Kochuvila S. (2023). Mobile Service Robot Path Planning Using Deep Reinforcement Learning. IEEE Access.

[97] Li P., Chen D., Wang Y., Zhang L., Zhao S. (2024). Path Planning of Mobile Robot Based on Improved TD3 Algorithm in Dynamic Environment. Heliyon.

[98] Weerakoon K., Sathyamoorthy A.J., Patel U., Manocha D. TERP: Reliable Planning in Uneven Outdoor Environments Using Deep Reinforcement Learning. Proceedings of the 2022 International Conference on Robotics and Automation (ICRA).

[99] Wang Y., He Z., Cao D., Ma L., Li K., Jia L., Cui Y. (2023). Coverage Path Planning for Kiwifruit Picking Robots Based on Deep Reinforcement Learning. Comput. Electron. Agric..

[100] Wei Y., Zheng R. Multi-Robot Path Planning for Mobile Sensing through Deep Reinforcement Learning. Proceedings of the IEEE INFOCOM 2021—IEEE Conference on Computer Communications.

[101] Mehmood A., Shaikh I.U.H., Ali A. (2021). Application of Deep Reinforcement Learning for Tracking Control of 3WD Omnidirectional Mobile Robot. Inf. Technol. Control.

[102] Fan F., Xu G., Feng N., Li L., Jiang W., Yu L., Xiong X. (2023). Spatiotemporal Path Tracking via Deep Reinforcement Learning of Robot for Manufacturing Internal Logistics. J. Manuf. Syst..

[103] Politi E., Stefanidou A., Chronis C., Dimitrakopoulos G., Varlamis I. (2024). Adaptive Deep Reinforcement Learning for Efficient 3D Navigation of Autonomous Underwater Vehicles. IEEE Access.

[104] Xiaoyang T., Zhang M., Zhang S.C. (2024). Traffic-Cognitive Slicing for Resource-Efficient Offloading with Dual-Distillation DRL in Multi-Edge Systems. arXiv.

[105] Chu S., Lin M., Li D., Lin R., Xiao S. (2025). Adaptive Reward Shaping Based Reinforcement Learning for Docking Control of Autonomous Underwater Vehicles. Ocean. Eng..

[106] Wang D., Yin H., Guo X., Wu J. (2024). Energy-Saving Optimization of Urban Rail Transit Timetable: A Deep Reinforcement Learning Approach. https://www.researchgate.net/publication/388478545_Energy-Saving_Optimization_of_Urban_Rail_Transit_Timetable_A_Deep_Reinforcement_Learning_Approach.

[107] Khaitan S. (2023). Exploring Reinforcement Learning Approaches for Safety Critical Environments. Master’s Thesis.

[108] Fan T., Long P., Liu W., Pan J., Yang R., Manocha D. (2020). Learning Resilient Behaviors for Navigation under Uncertainty. arXiv.

[109] Chen Y., Ji C., Cai Y., Yan T., Su B. (2024). Deep Reinforcement Learning in Autonomous Car Path Planning and Control: A Survey. arXiv.

[110] Günster J., Liu P., Peters J., Tateo D. (2024). Handling Long-Term Safety and Uncertainty in Safe Reinforcement Learning. arXiv.

[111] Aali M. (2025). Learning-Based Safety-Critical Control Under Uncertainty with Applications to Mobile Robots. Ph.D. Thesis.

[112] Kwon R., Kwon G. (2023). Safety Constraint-Guided Reinforcement Learning with Linear Temporal Logic. Systems.

[113] Wang J., Elfwing S., Uchibe E. (2021). Modular Deep Reinforcement Learning from Reward and Punishment for Robot Navigation. Neural Netw..

[114] Singh J. (2024). Robust AI Algorithms for Autonomous Vehicle Perception: Fusing Sensor Data from Vision, LiDAR, and Radar for Enhanced Safety. J. AI-Assist. Sci. Discov..

[115] Nissov M., Khattak S., Edlund J.A., Padgett C., Alexis K., Spieler P. ROAMER: Robust Offroad Autonomy Using Multimodal State Estimation with Radar Velocity Integration. Proceedings of the 2024 IEEE Aerospace Conference.

[116] Mitta N.R. (2024). AI-Enhanced Sensor Fusion Techniques for Autonomous Vehicle Perception: Integrating Lidar, Radar, and Camera Data with Deep Learning Models for Enhanced Object Detection, Localization, and Scene Understanding. J. Bioinform. Artif. Intell..

[117] Cheng W.-C., Ni Z., Zhong X., Wei M. (2024). Autonomous Robot Goal Seeking and Collision Avoidance in the Physical World: An Automated Learning and Evaluation Framework Based on the PPO Method. Appl. Sci..

[118] Yao C., Ge Y., Shi G., Wang Z., Yang N., Zhu Z., Wei H., Zhao Y., Wu J., Jia Z. (2024). TAIL: A Terrain-Aware Multi-Modal SLAM Dataset for Robot Locomotion in Deformable Granular Environments. IEEE Robot. Autom. Lett..

[119] Romanelli F. (2024). Multi-Sensor Fusion for Autonomous Resilient Perception. Ph.D. Thesis.

[120] Merveille F.F.R., Jia B., Xu Z., Fred B. (2024). Advancements in Sensor Fusion for Underwater SLAM: A Review on Enhanced Navigation and Environmental Perception. Sensors.

[121] Li Z., Zhou A. (2023). RDDRL: A Recurrent Deduction Deep Reinforcement Learning Model for Multimodal Vision-Robot Navigation. Appl. Intell..

[122] Huang X., Deng H., Zhang W., Song R., Li Y. (2021). Towards Multi-Modal Perception-Based Navigation: A Deep Reinforcement Learning Method. IEEE Robot. Autom. Lett..

[123] Hassan N.A. (2024). Big Data and Machine Learning in Autonomous Vehicle Navigation: Challenges and Opportunities. J. Appl. Cybersecur. Anal. Intell. Decis.-Mak. Syst..

[124] Tiwari R., Srinivaas A., Velamati R.K. (2025). Adaptive Navigation in Collaborative Robots: A Reinforcement Learning and Sensor Fusion Approach. Appl. Syst. Innov..

[125] Cole E.J., Thompson D.R., Nguyen J.T., Wright B.A. (2025). A Sensor-Fused Deep Reinforcement Learning Framework for Multi-Agent Decision-Making in Urban Driving Environments. Int. J. Eng. Adv..

[126] Raettig T.N. (2024). Heterogeneous Collaborative Robotics: Multi-Robot Navigation in Dynamic Environments. Master’s Thesis.

[127] Gao Y., Zhou D., Shen Y., Yang X. (2024). Dual Experience Replay-Based TD3 for Single Intersection Signal Control. J. Supercomput..

[128] Chen J. (2024). Reinforcement Learning and Swarm Intelligence for Cooperative Aerial Navigation and Payload Transportation. Ph.D. Thesis.

[129] Koradiya G. (2024). Reinforcement Learning Based Planning and Control for Robotic Source Seeking Inspired by Fruit Flies. Master’s Thesis.

[130] Wickenden Domingo À. (2024). Training Cooperative and Competitive Multi-Agent Systems. Bachelor’s Thesis.

[131] Dong L., He Z., Song C., Yuan X., Zhang H. (2024). Multi-Robot Social-Aware Cooperative Planning in Pedestrian Environments Using Attention-Based Actor-Critic. Artif. Intell. Rev..

[132] McClusky B. (2024). Dynamic Graph Communication for Decentralised Multi-Agent Reinforcement Learning. arXiv.

[133] Egorov V., Shpilman A. (2022). Scalable Multi-Agent Model-Based Reinforcement Learning. arXiv.

[134] Gronauer S., Diepold K. (2022). Multi-Agent Deep Reinforcement Learning: A Survey. Artif. Intell. Rev..

[135] Wang R. (2023). Towards Efficient Cooperation Within Learning Agents. Ph.D. Thesis.

[136] Chen R. Cooperative and Competitive Multi-Agent Deep Reinforcement Learning. Proceedings of the 2nd International Conference on Artificial Intelligence, Automation, and High-Performance Computing (AIAHPC 2022).

[137] Nekoei H., Badrinaaraayanan A., Sinha A., Amini M., Rajendran J., Mahajan A., Chandar S. Dealing with Non-Stationarity in Decentralized Cooperative Multi-Agent Deep Reinforcement Learning via Multi-Timescale Learning. Proceedings of the 2nd Conference on Lifelong Learning Agents.

[138] Oroojlooy A., Hajinezhad D. (2023). A Review of Cooperative Multi-Agent Deep Reinforcement Learning. Appl. Intell..

[139] Serra Gomez A. (2025). Motion Planning in Dynamic Environments with Learned Scalable Policies. Ph.D. Thesis.

[140] Cooperative Localization of UAVs in Multi-Robot Systems Using Deep Learning-Based Detection|AIAA SciTech Forum. https://arc.aiaa.org/doi/abs/10.2514/6.2025-1537.

[141] Jeong E., Gwak J., Kim T., Kang D.-O. Distributed Deep Learning for Real-World Implicit Mapping in Multi-Robot Systems. Proceedings of the 2024 24th International Conference on Control, Automation and Systems (ICCAS).

[142] Wang C. (2024). Robust AI Based Perception and Guidance for Autonomous Vehicles. Ph.D. Thesis.

[143] Muratore F., Ramos F., Turk G., Yu W., Gienger M., Peters J. (2022). Robot Learning from Randomized Simulations: A Review. Front. Robot. AI.

[144] Kang K., Belkhale S., Kahn G., Abbeel P., Levine S. Generalization through Simulation: Integrating Simulated and Real Data into Deep Reinforcement Learning for Vision-Based Autonomous Flight. Proceedings of the 2019 International Conference on Robotics and Automation (ICRA).

[145] Yavas M.U., Kumbasar T., Ure N.K. (2023). A Real-World Reinforcement Learning Framework for Safe and Human-like Tactical Decision-Making. IEEE Trans. Intell. Transp. Syst..

[146] Albuquerque P.L.F. (2019). Domain Adaptation in Unmanned Aerial Vehicles Landing Using Reinforcement Learning. Master’s Thesis.

[147] Wu J., Zhou Y., Yang H., Huang Z., Lv C. (2023). Human-Guided Reinforcement Learning with Sim-to-Real Transfer for Autonomous Navigation. IEEE Trans. Pattern Anal. Mach. Intell..

[148] Jang Y., Baek J., Jeon S., Han S. (2024). Bridging the Simulation-to-Real Gap of Depth Images for Deep Reinforcement Learning. Expert Syst. Appl..

[149] Zhao W., Queralta J.P., Westerlund T. Sim-to-Real Transfer in Deep Reinforcement Learning for Robotics: A Survey. Proceedings of the 2020 IEEE Symposium Series on Computational Intelligence (SSCI).

[150] Chukwurah N., Adebayo A.S., Ajayi O.O. (2024). Sim-to-Real Transfer in Robotics: Addressing the Gap between Simulation and Real-World Performance. JFMR.

[151] Muratore F. (2021). Randomizing physics simulations for robot learning. Ph.D. Thesis.

[152] Josifovski J., Malmir M., Klarmann N., Žagar B.L., Navarro-Guerrero N., Knoll A. (2022). Analysis of Randomization Effects on Sim2Real Transfer in Reinforcement Learning for Robotic Manipulation Tasks. arXiv.

[153] Dong Q., Zeng P., Wan G., He Y., Dong X. (2024). Kalman Filter-Based One-Shot Sim-to-Real Transfer Learning. IEEE Robot. Autom. Lett..

[154] Yu Y., Liu L. (2025). Neural Fidelity Calibration for Informative Sim-to-Real Adaptation. arXiv.

[155] Ju H., Juan R., Gomez R., Nakamura K., Li G. (2022). Transferring Policy of Deep Reinforcement Learning from Simulation to Reality for Robotics. Nat. Mach. Intell..

[156] Narkarunai Arasu Malaiyappan J., Mani Krishna Sistla S., Jeyaraman J. (2024). Advancements in Reinforcement Learning Algorithms for Autonomous Systems. Int. J. Innov. Sci. Res. Technol..

[157] Szolc H., Desnos K., Kryjak T. Tangled Program Graphs as an Alternative to DRL-Based Control Algorithms for UAVs. Proceedings of the 2024 Signal Processing: Algorithms, Architectures, Arrangements, and Applications (SPA).

[158] Mehta S. (2020). From AI Safety Gridworlds to Reliable Safety Unit Tests for Deep Reinforcement Learning in Computer Systems. Master’s Thesis.

[159] Yau H. On the Interpretability of Reinforcement Learning. https://www.surrey.ac.uk/events/20240626-interpretability-reinforcement-learning.

[160] Zhou Z., Liu G., Tang Y. (2024). Multiagent Reinforcement Learning: Methods, Trustworthiness, Applications in Intelligent Vehicles, and Challenges. IEEE Trans. Intell. Veh..

[161] Terven J. (2025). Deep Reinforcement Learning: A Chronological Overview and Methods. AI.

[162] Wang S., Zhang S., Zhang J., Hu R., Li X., Zhang T., Li J., Wu F., Wang G., Hovy E. (2025). Reinforcement Learning Enhanced LLMs: A Survey. arXiv.

[163] Hickling T., Zenati A., Aouf N., Spencer P. (2023). Explainability in Deep Reinforcement Learning, a Review into Current Methods and Applications. arXiv.

[164] Lu Y., Sun W., Sun M. (2022). Towards Mutation Testing of Reinforcement Learning Systems. J. Syst. Archit..

[165] Murad S.A., Muzahid A.J.M., Azmi Z.R.M., Hoque M.I., Kowsher M. (2022). A Review on Job Scheduling Technique in Cloud Computing and Priority Rule Based Intelligent Framework. J. King Saud. Univ.-Comput. Inf. Sci..

[166] Bao L., Humphreys J., Peng T., Zhou C. (2024). Deep Reinforcement Learning for Bipedal Locomotion: A Brief Survey. arXiv.

[167] Cai W., Cheng G., Kong L., Dong L., Sun C. (2023). Robust Navigation with Cross-Modal Fusion and Knowledge Transfer. arXiv.

[168] Kalenberg K., Müller H., Polonelli T., Schiaffino A., Niculescu V., Cioflan C., Magno M., Benini L. (2024). Stargate: Multimodal Sensor Fusion for Autonomous Navigation on Miniaturized UAVs. IEEE Internet Things J..

[169] Zhu F., Zhu Y., Lee V.C., Liang X., Chang X. (2021). Deep Learning for Embodied Vision Navigation: A Survey. arXiv.

[170] Hua J., Zeng L., Li G., Ju Z. (2021). Learning for a Robot: Deep Reinforcement Learning, Imitation Learning, Transfer Learning. Sensors.

